# Multi-omics and experimental validation identify USP54 as a prognostic deubiquitinase promoting pancreatic ductal adenocarcinoma progression within the immune microenvironment

**DOI:** 10.3389/fimmu.2026.1791707

**Published:** 2026-03-18

**Authors:** Zibo Yuan, Zhiwei Yu, Qiuran Xu, Dongsheng Huang, Di Cui

**Affiliations:** 1 The Qingdao Medical College of Qingdao University, Qingdao, China; 2 Zhejiang Key Laboratory of Tumor Molecular Diagnosis and Individualized Medicine, Zhejiang Provincial People’s Hospital (Affiliated People’s Hospital), Hangzhou Medical College, Hangzhou, China; 3 General Surgery, Cancer Center, Department of Gastrointestinal and Pancreatic Surgery, Zhejiang Provincial People’s Hospital (Affiliated People’s Hospital), Hangzhou Medical College, Hangzhou, China; 4 School of Public Health, Hangzhou Medical College, Hangzhou, China

**Keywords:** deubiquitinase, immune microenvironment, pancreatic ductal adenocarcinoma, single-cell, spatialomics, USP54

## Abstract

**Background:**

Pancreatic ductal adenocarcinoma (PDAC) is a highly lethal malignancy with a complex tumor ecosystem that contributes to its progression. Deubiquitinases (DUBs) are vital regulators in cancer. However, the overall activity of DUBs and their role in driving PDAC progression within immune microenvironment remain largely unknown.

**Methods:**

We employed an integrative multi-omics strategy combining machine learning (ML) on bulk transcriptomic data, single-cell RNA sequencing and spatial transcriptomic profiling. We applied Coxnet and Fuzzy SVM for prognostic modeling, inferCNV for malignant cell identification, SCENIC for transcription factor regulon analysis, LIANA^+^ for inferring inter−cellular communication networks and cell2location for spatial deconvolution. USP54 expression was detected by real-time quantitative PCR, western blotting and immunohistochemistry. USP54 function was validated through *in vitro* and *in vivo* assays.

**Results:**

ML-based pathway analysis revealed post−translational modification as a major prognostic category, within which elevated DUBs activity emerged as an independent adverse prognostic factor. At the single−cell level, USP54 was upregulated along the trajectory of malignant ductal cells and correlated with an inflamed tumor microenvironment. Cell−cell communication analysis predicted signaling from monocytes/macrophages to tumor cells via the THBS1−integrin ligand−receptor pair. This immune−derived signaling potentially converged on KLF5−positive tumor cells, with KLF5 identified as a putative transcriptional activator of USP54. Spatial transcriptomics validated the co−localization of USP54 expression, elevated DUB activity, and KRAS signaling within specific tumor niches adjacent to THBS1−enriched immune regions. High USP54 expression was frequently observed in PDAC tissues and associated with poor patient survival. More importantly, in both BxPC-3 and PANC-1 cell lines, USP54 knockdown suppressed cell proliferation and metastasis, whereas its overexpression enhanced these malignant phenotypes. Subcutaneous xenograft growth and tail vein injection experiments validated these findings *in vivo*.

**Conclusions:**

Our comprehensive multi-omics analysis and experimental validation identify the deubiquitinase USP54 as a novel promoter of PDAC progression within a spatially organized tumor−immune microenvironment. These findings suggest USP54 as both a candidate prognostic biomarker and a potential therapeutic target for this lethal malignancy.

## 1 Introduction

Pancreatic ductal adenocarcinoma (PDAC) is an aggressive malignancy with a five-year survival rate persistently below 10% (1). This poor prognosis is largely attributable to late diagnosis, rapid disease progression, and limited therapeutic efficacy (2). A deeper understanding of the molecular mechanisms driving PDAC progression, especially the crosstalk between cancer cells and the immune microenvironment, along with the identification of reliable biomarkers for prognosis, is urgently needed to develop more effective therapeutic strategies.

In recent years, with the rapid development of high-throughput sequencing technologies, multi-omics technologies such as bulk transcriptomics, single-cell RNA sequencing (scRNA-seq), and spatial transcriptomics have provided powerful means for comprehensively characterizing tumor heterogeneity and cell-cell interactions in the tumor microenvironment, playing a crucial role in the discovery of tumor biomarkers (3). In PDAC research, these technologies have successfully identified the intrinsic molecular subtypes of tumors and revealed various novel cell-cell communication patterns (4). Machine learning (ML) methods have further improved the performance of prognostic prediction models by systematically identifying gene expression features significantly associated with patient survival. For example, some studies have systematically evaluated more than 100 machine-learning algorithms to construct the optimal gene expression risk scoring model for PDAC (5). However, models that use single genes as the analysis unit have inherent limitations: due to the neglect of the functional synergy between genes and the biological coordination mechanisms at the pathway level, their generalization ability in different populations is often insufficient (6). The above issues suggest that there is an urgent need to develop prognostic models that integrate pathway-level information to enhance the biological interpretability and clinical applicability of the models.

Although molecular typing studies have significantly deepened our understanding of PDAC heterogeneity (7), the potential value of specific functional pathways in prognosis assessment still lacks systematic exploration. Post-translational modifications (PTMs) of proteins, especially the processes of ubiquitination and deubiquitination, have been proven to be one of the key mechanisms regulating cancer progression (8, 9). Deubiquitinases (DUBs) play a central role in cell signal transduction by regulating the stability and functional activity of target proteins, and their abnormal expression or dysfunction is closely related to the occurrence and development of various malignant tumors (10, 11). However, there is still a lack of systematic research on the activity profiles of DUBs and their clinical significance in PDAC. Although USP54 has been implicated in several cancer types, including colorectal cancer and lung adenocarcinoma, its functional role in PDAC remains unexplored (12, 13).

In this study, we employed a pathway-based machine learning framework to systematically identify biological processes associated with the prognosis of PDAC. The analysis results show that PTM is a core biological process significantly associated with the invasiveness of PDAC, and an overall increase in the activity of DUBs can serve as an independent predictor of poor prognosis. At the single-cell level, this DUB activity and USP54 expression were enriched within malignant ductal cells and correlated with an inflamed tumor microenvironment. Spatial analysis revealed that its expression was localized to tumor cell niches adjacent to immune regions enriched for specific signals such as THBS1. Functional experiments further established the deubiquitinase USP54 as a novel regulatory factor in the progression of PDAC. Our findings suggest USP54 as a candidate prognostic biomarker and potential therapeutic target in PDAC.

## 2 Materials and methods

### 2.1 Data acquisition

Bulk RNA-sequencing data and corresponding clinical information for pancreatic adenocarcinoma (PAAD) were obtained from The Cancer Genome Atlas (TCGA) database, comprising 142 patients. Additional bulk transcriptomic datasets were downloaded from the Gene Expression Omnibus (GEO), including GSE62452 (61 adjacent non-tumor and 69 PDAC samples) (14), GSE28735 (45 adjacent non-tumor and PDAC paired samples) (15), and GSE85916 (80 PDAC samples with survival time). Single-cell RNA-sequencing datasets were obtained from GSE212966 (6 adjacent and 6 PDAC samples) and GSE197177 (1 adjacent and 3 PDAC samples) (16, 17), while spatial transcriptomic data were acquired from GSE233293 (3 PDAC samples) (18).

### 2.2 Construction of pathway-level ssGSEA features

To aggregate gene-level expression into pathway-level features, we performed single-sample gene set enrichment analysis (ssGSEA) using the GSEApy implementation based on the Gene Ontology Biological Process (GO BP) collection from the Human MSigDB resource (19, 20). For TCGA-PAAD (training set) and the combined GEO cohorts GSE28735, GSE62452, and GSE85916 (external sets), this yielded a sample-by-pathway matrix of normalized enrichment scores (NES) used as the primary feature matrix for survival modeling. GO BP gene sets were chosen because they capture coordinated biological processes and provide functionally interpretable, widely curated signatures that enhance biological interpretation and cross-cohort comparability.

### 2.3 Train-validation split and handling of external test cohort

For the main TCGA-based cohort, we constructed a pathway-by-sample NES matrix and aligned it with the corresponding clinical table. We stipulated that samples with follow-up times close to 1 year, 3 years, and 5 years were represented in either the training set or the validation set. The remaining samples were randomly divided into a training set and an internal validation set at a ratio of 7:3, and stratified sampling was implemented as much as possible according to the occurrence of events during the division process.

### 2.4 Feature pre-selection

Feature selection was performed exclusively within the training set to reduce dimensionality and avoid information leakage. As the primary pre-selection strategy, univariate Cox proportional hazards regression was applied to each candidate pathway by fitting a Cox model with NES as the sole covariate using CoxPHFitter. Wald test P-values were obtained and adjusted for multiple testing using the Benjamini–Hochberg false discovery rate (FDR) procedure, and pathways with FDR ≤ 0.05 were retained for downstream multivariable modeling. This univariate Cox–based pre-selection was applied uniformly across all predictive models to ensure a transparent and model-agnostic screening process without additional correlation-based redundancy removal. As a supplementary analysis, feature robustness was further assessed using stability selection based on an elastic-net–penalized Cox model (CoxnetSurvivalAnalysis), which was repeatedly fitted on 1,000 bootstrap resamples of 70% of the training data (with replacement) using a fixed mixing parameter (l1_ratio = 0.1). A pathway was considered selected in a given resample if it exhibited a non-zero coefficient for any regularization parameter, and the selection frequency across resamples was used as a measure of feature stability.

### 2.5 Model development and hyperparameter tuning

All models were trained on ssGSEA-derived normalized enrichment score (NES) features from the training set, after standardization with a scaler fitted on the training data and applied to all cohorts. We benchmarked four survival algorithms: Random Survival Forest (RSF), Gradient Boosting Survival Analysis (GBSA), fast kernel survival support vector machine (FSVM), and an XGBoost Cox model.

Hyperparameters for each model were tuned separately using Optuna with a tree-structured Parzen estimator (TPE). Model selection was based on 5-fold cross-validation in the training cohort, using Harrell’s concordance index (C-index) as the optimization objective; for each algorithm, the hyperparameter set with the highest mean cross-validated C-index was chosen and the corresponding model was then refitted on the full training set.

### 2.6 Model evaluation and external validation

The final models were first evaluated on the internal validation set and then on each external test cohort. Model discrimination was quantified using C-index. To assess time-dependent performance, we additionally calculated time-dependent area under the curve (AUC) values at 1, 3, and 5 years.

### 2.7 Model-based feature importance

For the FSVM model, feature importance was assessed using SHapley Additive exPlanations (SHAP) to provide a *post hoc*, model-agnostic interpretation of pathway contributions. Given the non-linear and kernel-based nature of FSVM, SHAP was applied to approximate the marginal contribution of each pathway feature to the model’s risk predictions rather than to infer causal effects. Specifically, SHAP values were computed at the individual patient level based on the fitted FSVM model, quantifying the contribution of each pathway ssGSEA score to the predicted survival risk for that patient. To summarize global feature importance, SHAP values were averaged across all patients for each pathway, yielding a mean SHAP value per feature. Pathways with positive mean SHAP values were interpreted as contributing to higher predicted mortality risk, whereas pathways with negative mean SHAP values were considered protective and associated with lower predicted death risk. We acknowledge that, as with other additive feature attribution methods, SHAP values may be influenced by correlation among pathway features, which is inherent to GO biological process annotations. Therefore, SHAP-based interpretations were used to highlight dominant and recurrent prognostic patterns rather than to establish unique or independent pathway effects. Accordingly, SHAP results were interpreted in conjunction with the statistical feature selection procedure and overall model performance, and were not used as the sole basis for biological inference.

### 2.8 Single-cell RNA-seq preprocessing and quality control

Raw gene-barcode matrices (10x Genomics format) were imported into Seurat (V5) to create sample-specific objects with a minimum of 200 detected genes and 50 cells per gene (21). For each sample, we performed quality control by retaining cells with 200–10,000 detected genes and excluding cells with >20% mitochondrial and >15% hemoglobin transcript proportion. Putative doublets were identified using scDblFinder (run 10 times), and only cells consistently classified as doublets across iterations were removed (22). To reduce ambient RNA and contamination, we applied scCDC (23) and decontX (24).

For integrated analysis, we used Scanpy (25) (library-size normalization to 10,000 counts, log-transformation, and selection of 2,000 highly variable genes using the Seurat v3 flavor), followed by scaling, PCA, and batch correction across samples with batch balanced k-nearest neighbors (BBKNN) (26); Uniform Manifold Approximation and Projection (UMAP) embeddings were then computed, and Leiden clustering was performed over a range of resolutions (0.5–1.4), with the resolution maximizing the silhouette score in UMAP space chosen as the final clustering granularity. Cell types were manually annotated based on canonical marker genes with reference to the CellMarker 2.0 database (27).

### 2.9 Tumor cell recognition and deconvolution

Copy-number variation (CNV) profiles were inferred with infercnvpy (28), using all annotated immune and non-malignant stromal populations (CD8^+^/CD4^+^ T cells, B cells, NK cells, Tregs, neutrophils, mast cells, plasma cells, monocytes/macrophages, endothelial cells, Schwann cells) as the reference and a sliding window of 100 genes on normalized expression values. We then performed PCA, built a neighbor graph, and ran Leiden clustering across a range of resolutions (0.2-2.0), selecting the resolution that maximized the silhouette score in UMAP space as the final CNV cluster partition, and computed per-cell CNV scores. To detect focal events in key driver genes (*CDKN2A, CDKN2B, TP53, KRAS*) (29), we calculated robust Z-scores for the infercnvpy gene-value layer relative to the reference (median and median absolute deviation per gene) and thresholded these Z-scores to classify each gene in each cell as copy-number gain, loss, or neutral, storing the resulting categorical calls.

Importantly, CNV inference was performed exclusively at the single-cell level. Bulk-level analyses in TCGA were instead used for complementary purposes, rather than CNV inference per se. Specifically, cell-type deconvolution was conducted on bulk RNA-seq profiles using BayesPrism (28) to estimate sample-level cellular composition. The inferred cell-type fractions were then incorporated into univariable and multivariable Cox proportional hazards models to identify cell populations whose abundance was significantly associated with overall survival.

### 2.10 Inference of PTM signatures in single-cell and bulk cohorts

In the single-cell data, post-translational modification (PTM)-related gene set activity scores were computed using AUCell as implemented in decoupler (30) (**Supplementary Table 1**); the resulting AUCell scores were then used to rank PTM signatures for each cell type using a Wilcoxon rank-sum test, and the top three positively enriched PTM categories per cell type were retained. In the TCGA cohort, PTM-related gene set activity was quantified by ssGSEA, followed by univariable and multivariable Cox regression analyses to identify prognosis-associated PTM gene sets (31).

### 2.11 Differentially expressed genes

Differential expression analysis at the single-cell level was performed using omicverse (32), comparing Tumor ductal cells versus Normal ductal cells with a two-sided t-test; genes with *q* value <0.05 and absolute log2 fold change >1 were defined as differentially expressed genes (DEGs). Differential expression analysis of bulk transcriptomic data was performed using the limma (33), and genes with an absolute log_2_ fold change >0.5 and Benjamini–Hochberg adjusted P-value (adj.P.Val) < 0.05 were considered differentially expressed.

### 2.12 Single-cell trajectory and pseudo-time analysis

For single-cell trajectory analysis, we first applied CytoTRACE2 to infer differentiation potency and identify putative progenitor and terminal cell populations (34). We then constructed pseudotime trajectories using scTour and diffusion pseudotime (DPT) (35, 36), and used MAGIC-based imputation to denoise gene expression profiles along the inferred trajectories (37). The start and end states were algorithmically determined by the pseudotime framework based on global data structure, without manual predefinition. After trajectory construction, we validated the biological relevance of the inferred endpoints by examining established lineage markers, confirming consistency with known cellular transitions. Based on these pseudotime orderings, we further examined dynamic changes in selected gene expression and pathway activity scores over pseudotime to characterize temporal rewiring of cellular states.

### 2.13 Transcription factor analysis

Gene regulatory network inference and regulon activity scoring were performed with pySCENIC (38). Based on the resulting regulon activity matrix, we then identified celltype-specific transcription factor markers.

### 2.14 Cell-cell communication analysis and causal network inference

Cell-cell communication was inferred with LIANA^+^ by meta-aggregating six methods (SingleCellSignalR, Connectome, CellPhoneDB, NATMI, logFC, and CellChat) with 1,000 permutations (39); significant ligand-receptor pairs were defined as those with all pvals < 0.05 and ligand-receptor logfc > 1. For causal network inference, we built a signed, directed prior knowledge network from OmniPath (40) (SIGNOR interactions with consensus directionality, encoded as +1 for activation and -1 for inhibition), converted it into a CORNETO graph, and used node-level input/output scores as constraints in a CarnivalFlow optimization problem solved with the HiGHS solver; edges with |edge_value| > 0.5 were retained and visualized as the final context-specific signaling network.

### 2.15 Promoter motif analysis

Transcription start site (TSS) coordinates and gene annotations were retrieved from Ensembl (GRCh38/hg38) via pybiomart, and promoter regions were defined as -1 kb to +100 bp around the TSS; corresponding genomic sequences were extracted from the hg38 reference genome using pyfaidx. Position weight matrices for candidate transcription factors were obtained from JASPAR 2024 and converted to MEME format, and promoter sequences were scanned with FIMO (MEME suite) using a significance threshold of p < 0.01 to identify motif hits, which were then mapped back to target genes based on genomic overlap.

### 2.16 Weighted gene co-expression network analysis

To identify co-expressed gene modules that are highly correlated with clinical/phenotypic traits across the genome, thereby uncovering potential key pathways and pivotal genes, we used WGCNA (41). Samples and genes with poor quality were removed and outlier samples were excluded based on hierarchical clustering. A soft-thresholding power was selected using a scale-free topology criterion (R² = 0.85), and an unsigned network was constructed with minModuleSize = 30 and mergeCutHeight = 0.25 to identify gene modules. Module eigengenes (MEs) were calculated and correlated with clinical traits to generate a module-trait correlation heatmap, and we further examined the relationship between gene significance (GS) and module membership (MM) within each module to prioritize hub genes.

### 2.17 Single gene analysis

We first calculated the log_2_(TPM+1) level of gene expression based on TCGA-PAAD and other TCGA tumor cohorts, and plotted differential expression maps between tumors and adjacent normal tissues in pancreatic cancer across cancer types. In the paired GSE28735 and GSE62452 cohorts, the paired test was used to assess the differential expression of USP54 between PDAC and adjacent tissues. Subsequently, patients were divided into high-expression and low-expression groups based on the optimal threshold for USP54 expression in the TCGA-PAAD cohort. Kaplan-Meier curves and log-rank tests were used to assess differences in overall survival, and the distribution differences between the two groups in terms of age and clinical stage (T, N stages and WHO histological grade) were compared. To assess the relationship between USP54 expression and the tumor immune microenvironment, we performed correlation analysis on the proportions of various immune infiltrating cells (such as B cells, CD8^+^/CD4^+^ T cells, macrophages, neutrophils, and dendritic cells) and tumor purity by TIMER2.0 (42). We combined clinical data and gene expression levels to explore prognostic factors through univariate and multivariate retrospective analysis. At the single-cell level, we divided tumor ductal cells into three groups (Low/Mid/High) based on USP54 magic expression levels, and compared functional scores (Kruskal–Walli’s test) among the three groups, including deubiquitinase (DUB)-related scores and KRAS signaling pathway activity. Simultaneously, we screened for USP54-high expression-related genes through differential expression analysis. Further Hallmark pathway enrichment analysis was performed on these differentially expressed genes to elucidate key biological pathways related to USP54.

### 2.18 Spatial transcriptome analysis

Visium PDAC sections (PDAC1-3) were processed with OmicVerse by normalizing to 10,000 counts per spot, and selecting 2,000 spatially variable genes. GraphST was then applied independently to each section to learn graph-based spatial embeddings and perform Leiden clustering (43). We used cell2location to project single-cell–defined cell type signatures onto the Visium spatial transcriptomics sections, thereby estimating the spatial abundance of each cell type at spot resolution (44). We applied COMMOT to infer spatially resolved ligand–receptor communication, using the mapped cell-type abundances and spatial coordinates to identify ligand–receptor pairs whose inferred interaction strength was enriched between neighboring spots, thereby highlighting putative communication axes in the tumor microenvironment (45).

### 2.19 Cell culture and transfection

Human PDAC cell lines BxPC-3 and PANC-1 were obtained from the Cell Bank of the Chinese Academy of Sciences. BxPC-3 cells were maintained in RPMI-1640 medium (VivaCell, C3010-0500), and PANC-1 cells were cultured in DMEM (VivaCell, C3113-0500). All media were supplemented with 10% fetal bovine serum and 1% penicillin–streptomycin, and cells were incubated at 37 °C in a 5% CO_2_ atmosphere.

For gene perturbation studies, siRNAs and shRNAs targeting USP54, a full-length USP54 overexpression plasmid, and USP54 lentiviral constructs were obtained from GenePharma, respectively. Transient transfection was performed using liposomal transfection reagent (Yeasen, 40802ES03) according to the manufacturer’s instructions. Sequences of siRNAs and shRNAs are provided in **Supplementary Table 2**.

### 2.20 Real-time quantitative pcr

RT-qPCR was performed as previously described (46). Primer sequences are listed in **Supplementary Table 3**.

### 2.21 Western blot

WB was conducted according to previous protocols (46). Antibody details are provided in **Supplementary Table 4**.

### 2.22 Cell counting kit-8 and colony formation assays

For Cell counting kit-8 (CCK-8) assays, BxPC-3 and PANC-1 cells were seeded in 96-well plates at optimized densities. After cell attachment, CCK-8 reagent (Yeasen, 40203ES80) was added to each well at designated time points, and the absorbance at 450 nm was measured. In the colony formation assay, cells were seeded at a low density in 6-well plates and cultured in complete medium for 7–14 days. After colony formation, the colonies were rinsed with PBS, fixed with 4% paraformaldehyde, stained with 2% crystal violet, rinsed with distilled water and air-dried. Finally, imaging and quantitative analysis are performed.

### 2.23 Wound healing and transwell assays

Briefly, for wound healing assays, a scratch was introduced into a confluent cell monolayer, and wound closure was monitored over time. For transwell assays, cells were seeded in the upper chamber of transwell inserts, and migratory cells on the lower membrane surface were stained and counted after an appropriate incubation period. Wound healing and transwell assays were performed following standard protocols (47).

### 2.24 Tumor sphere formation assay

Tumor sphere formation assay was conducted according to previous protocols (46).

### 2.25 Immunohistochemistry

Tissue specimens were fixed, paraffin-embedded, and sectioned. Antigen retrieval was performed, followed by blocking of endogenous peroxidase. Sections were incubated with primary antibody overnight at 4 °C, washed with PBS, and then incubated with secondary antibody for 1 hour at room temperature. Immunocomplexes were visualized using a DAB detection kit (ZSGB-BIO, PV8000) with a 5-minute chromogenic reaction. Counterstaining was carried out with hematoxylin, after which sections were dehydrated through graded ethanol, cleared in xylene, and mounted with neutral balsam for digital scanning. Staining was evaluated independently by two pathologists based on both intensity and extent. Any discrepancies were resolved by joint re-review under identical microscopic conditions.

### 2.26 Mouse models

To establish tumor models, PANC-1 cells with stable USP54 knockdown or overexpression were used. Female BALB/c nude mice (5–6 weeks old), supplied by Hangzhou Medical College. Nude mice were randomly assigned to experimental groups using a computer-generated random number table and allocated to two independent experiments: a subcutaneous implantation model and an intravenous injection model. Both tumor volume and body weight measurements were conducted in a blinded manner. The pre-established exclusion criteria were as follows: mice exhibiting severe systemic illness or a body weight loss exceeding 15% from baseline during the experiment were excluded. Additionally, nude mice that died from non-tumor-related accidental causes were not included in the final analysis. It is noteworthy that mice with baseline tumor volumes less than 50 mm³ were not excluded, as the shUSP54 knockdown group demonstrated a significant tumor growth inhibition effect, inherently resulting in smaller tumor volumes compared to the control group. Excluding small-volume tumor specimens would selectively eliminate key data from the shUSP54 group, introducing selection bias and consequently compromising the accuracy of efficacy evaluation. Ultimately, all mice meeting the criteria were included in the final statistical analysis. For the subcutaneous model, 25 mice were randomly assigned to 5 groups (5 mice per group) and each received an injection of 5×10^6^ cells. Tumor growth was monitored by periodic measurement of length (L) and width (W) with a digital caliper; volume (V) was subsequently calculated as 0.52 × L × W². Concurrently, in the intravenous model, 15 mice were randomized into 5 groups (3 mice per group) and injected via the tail vein with 4×10^6^ cells per mouse. At the end of the study, all animals were humanely euthanized. The subcutaneous tumors were excised and measured for volume and weight, while the lungs from the intravenous model were collected for quantification of metastatic nodules.

## 3 Results

**Figure 1:**
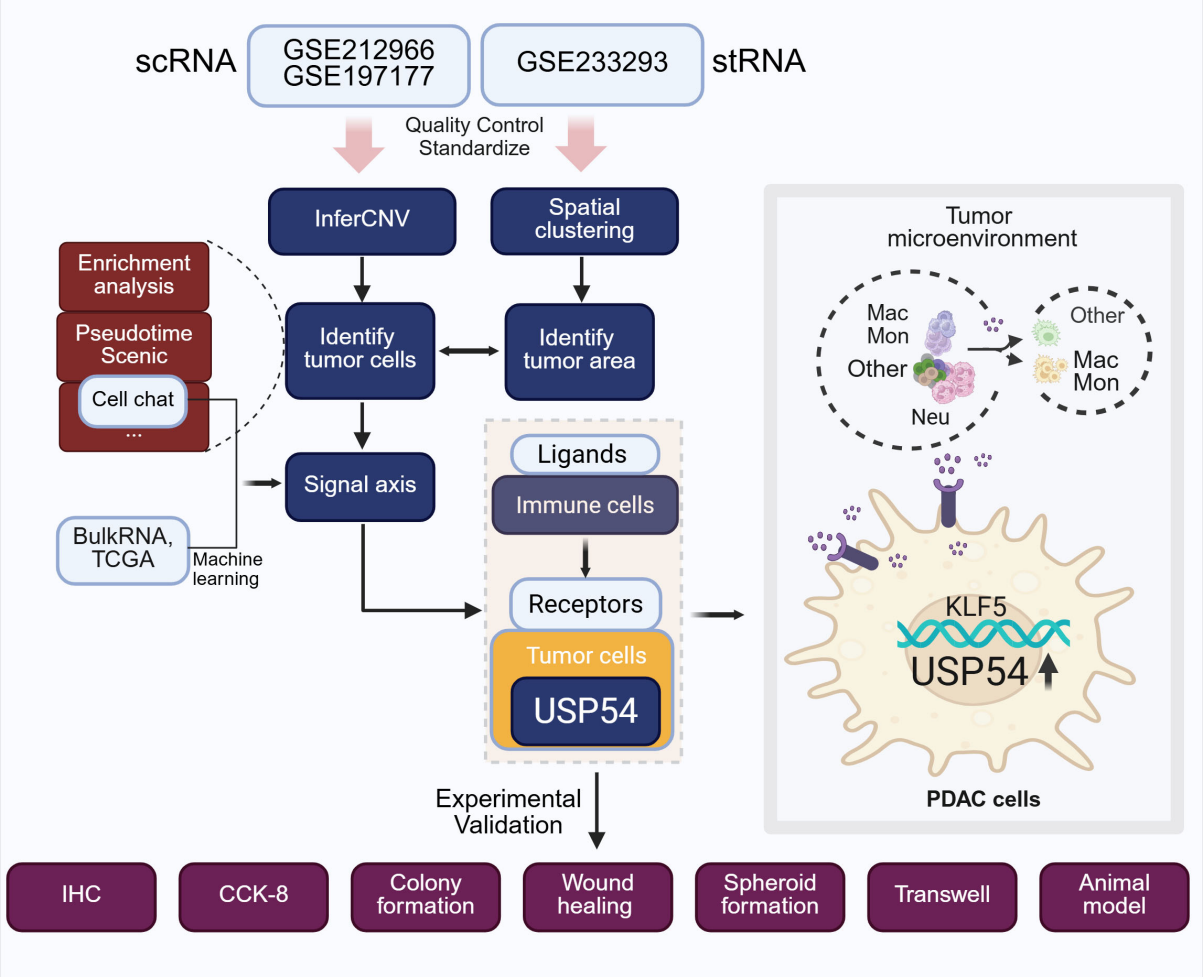
Overall design process.

Overall Design Process as **Figure 1**.

### 3.1 Adverse prognosis with elevated deubiquitinase activity in the machine learning identified post−translational modification category

**Figure 2:**
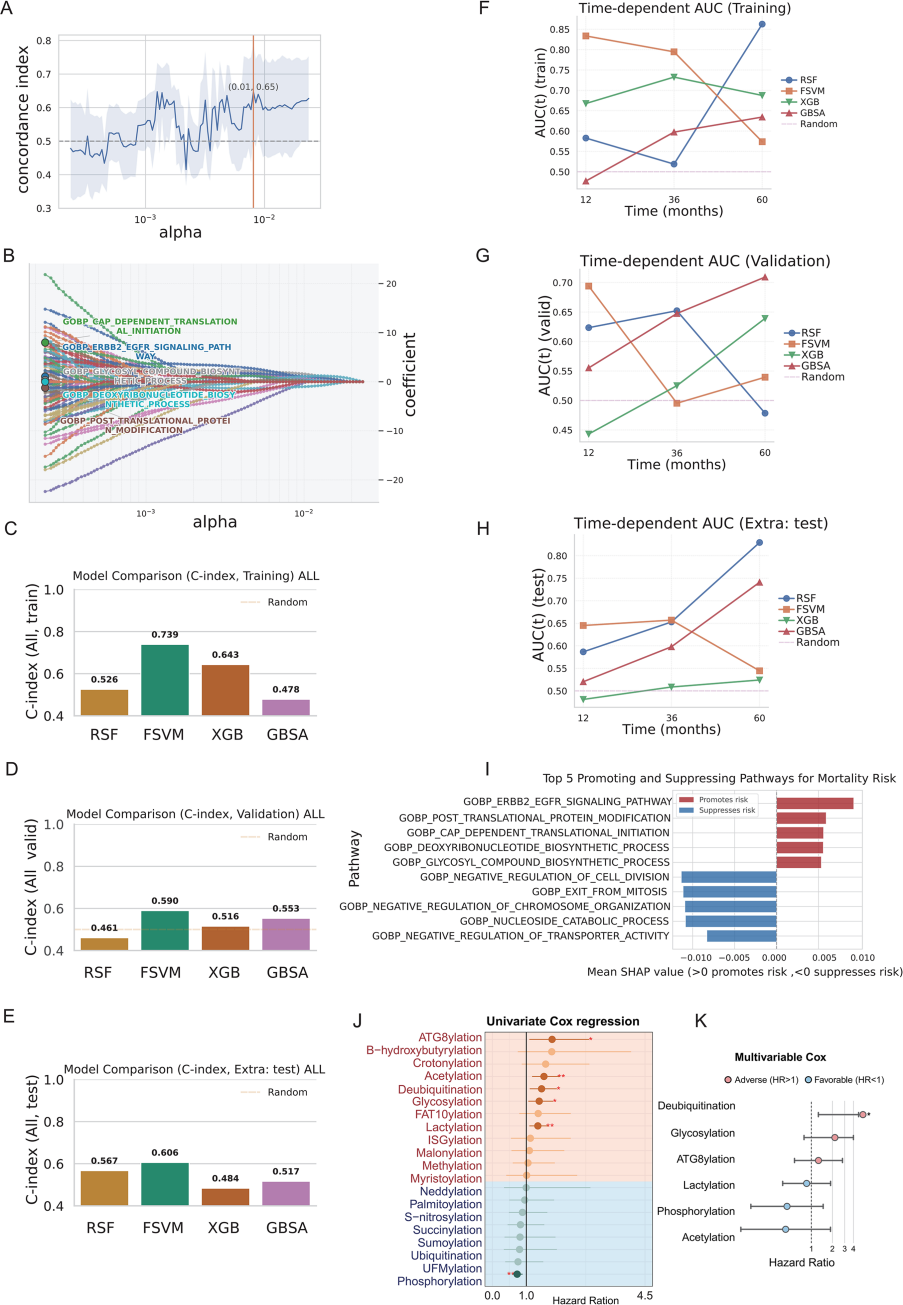
Pathway-level survival modeling. **(A)** Hyper-parameter tuning of the Cox-based model. Mean cross-validated Harrell’s C-index across alphas (solid line, shaded area = ±1 SD); the vertical line marks the selected alpha. **(B)** Coefficient paths of the elastic-net Cox model (Coxnet) across the alpha grid, illustrating shrinkage of representative GO biological process pathways as penalization increases. **(C–E)** Comparison of discrimination performance (C-index) for RSF, FSVM, XGBoost Cox (XGB) and GBSA in the training cohort **(C)**, internal validation cohort **(D)** and external test cohort **(E)**. The dashed line indicates the expected C-index for a random model. **(F–H)** Time-dependent AUCs at 12, 36 and 60 months for the same four models in the training **(F)**, validation **(G)** and external test **(H)** cohorts. **(I)** Top five pathways with the strongest risk-promoting (red) and risk-suppressing (blue) effects, ranked by mean SHAP values from the best-performing model. **(J)** Univariate Cox regression of post-translational modification (PTM) activity scores, shown as hazard ratios (HR) with 95% confidence intervals; PTMs with HR > 1 are associated with increased mortality risk. **(K)** Multivariable Cox model including selected PTM scores, displaying adjusted HRs and 95% confidence intervals.

To systematically identify biological pathways critically associated with PDAC prognosis, we employed a multi-step machine learning (ML) framework. We first optimized the regularization strength of the Coxnet model and identified an α of 0.01 as yielding the highest cross-validated C-index (0.65), with relatively stable performance across nearby α values (**Figure 2A**). The corresponding coefficient paths showed that a subset of GO BP signatures were retained (**Figure 2B**). Across all four algorithms, FSVM achieved the best overall discrimination: in the training, internal validation and external test cohorts, the FSVM model reached C-indices of 0.739, 0.590 and 0.606, respectively, clearly outperforming RSF, XGBoost and GBSA, which all remained close to the random baseline of 0.5 (**Figures 2C–E**; **Supplementary Figure 1**). Time-dependent ROC analysis further confirmed this advantage, with FSVM showing superior or comparable AUCs at 12, 36 and 60 months in the training, validation and external test cohorts (**Figures 2F–H**). Using SHAP values derived from the final FSVM model, we next quantified the contribution of each pathway signature to mortality risk. Pathways related to ERBB2-EGFR signaling and post-translational protein modification were identified as top risk factors (positive SHAP values), while processes that restrain cell division were protective (negative SHAP values) (**Figure 2I**). We then focused specifically on enzyme activity−based scores representing post−translational modification (PTM). Univariable Cox regression confirmed that several PTM−related activity scores were significantly associated with overall survival (**Figure 2J**). More importantly, a higher deubiquitinase (DUB) activity score remained an independently predictor of adverse prognosis in the multivariable Cox model (**Figure 2K**).

### 3.2 Single-cell atlas of PDAC

**Figure 3:**
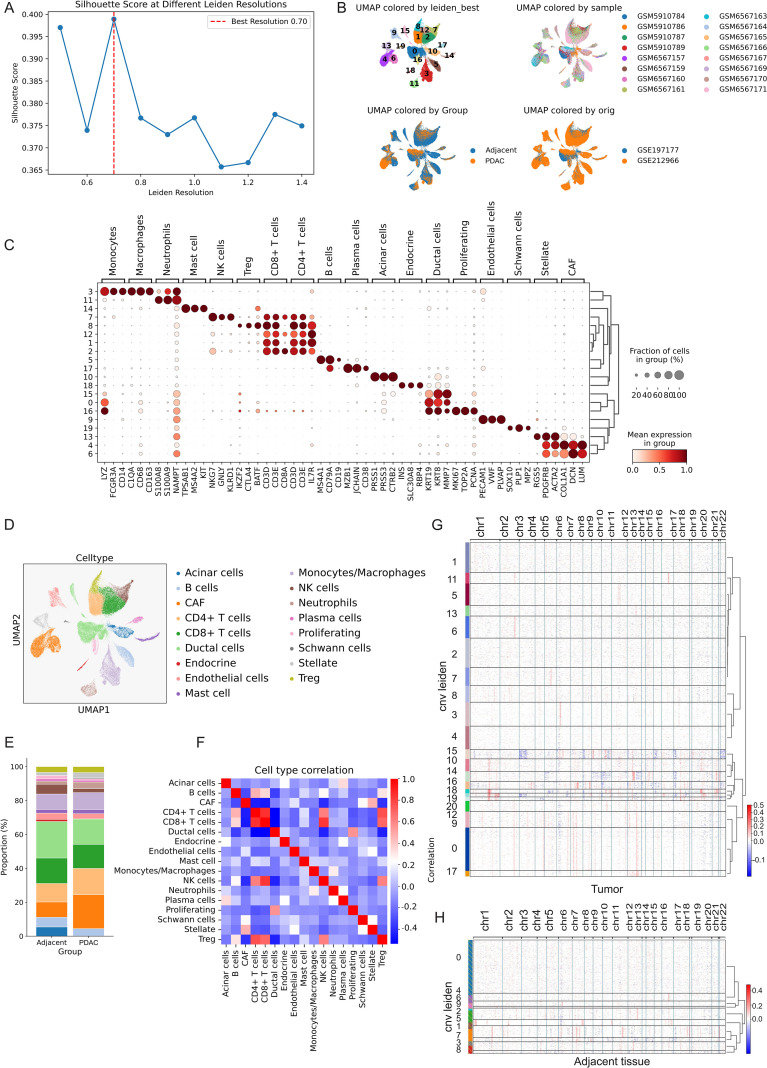
Single-cell atlas of PDAC and adjacent tissue and inference of malignant ductal populations. **(A)** Silhouette score across Leiden resolutions used to select the optimal clustering granularity; the red dashed line marks the chosen resolution (0.70). **(B)** UMAP embedding of all cells colored by optimal Leiden clusters, by sample, by group, and by dataset of origin. **(C)** Dot plot of canonical marker genes across Leiden clusters, showing the fraction of cells expressing each gene (dot size) and the average normalized expression (color scale). **(D–F)** UMAP visualization of annotated cell types **(D)**, stacked bar plot of cell‐type composition in adjacent versus PDAC samples **(E)**, and pairwise Spearman correlation heatmap of cell‐type proportions across samples **(F)**. **(G, H)** Inferred large‐scale copy number variation (CNV) profiles along autosomes for ductal and proliferating cells derived from tumor **(G)** and adjacent tissue **(H)**, with rows representing inferred CNV segments and columns representing individual cells.

We next constructed an integrated single‐cell atlas of PDAC and matched adjacent tissues. Silhouette-guided tuning of the Leiden resolution identified 0.70 as the optimal granularity, yielding well-separated clusters in UMAP visualization (**Figures 3A, B**). Using canonical marker genes (**Figures 3C, D**), we identified and annotated 17 distinct cell clusters, including acinar cells, ductal cells, cancer-associated fibroblasts (CAFs), stellate cells, endothelial cells, endocrine cells, B cells, CD4^+^ and CD8^+^ T cells, NK cells, plasma cells, neutrophils, monocytes/macrophages, Tregs, Schwann cells, mast cells and proliferating cells, collectively representing the major tumor, stromal, and immune compartments within the PDAC microenvironment. Quantitative analysis revealed that the proportion of stellate cells, CAFs, Schwann cells, CD4^+^ T cells, neutrophils and monocytes/macrophages were increased in tumor samples compared to adjacent samples (**Figure 3E**), consistent with the characteristic features of PDAC, including complex immune infiltration and perineural invasion (48–50). Cell-type correlations highlighted accuracy of annotations (**Figure 3F**). To confirm malignant transformation at the genomic level, we inferred large-scale CNV profiles of ductal cells. Ductal clusters within the tumor region exhibited extensive chromosomal gains and losses, whereas ductal cells from adjacent tissues showed largely flat CNV patterns (**Figures 3G, H**), supporting the designation of the tumor ductal cluster as the malignant compartment used for downstream analyses.

### 3.3 Precise isolation of malignant cells

**Figure 4:**
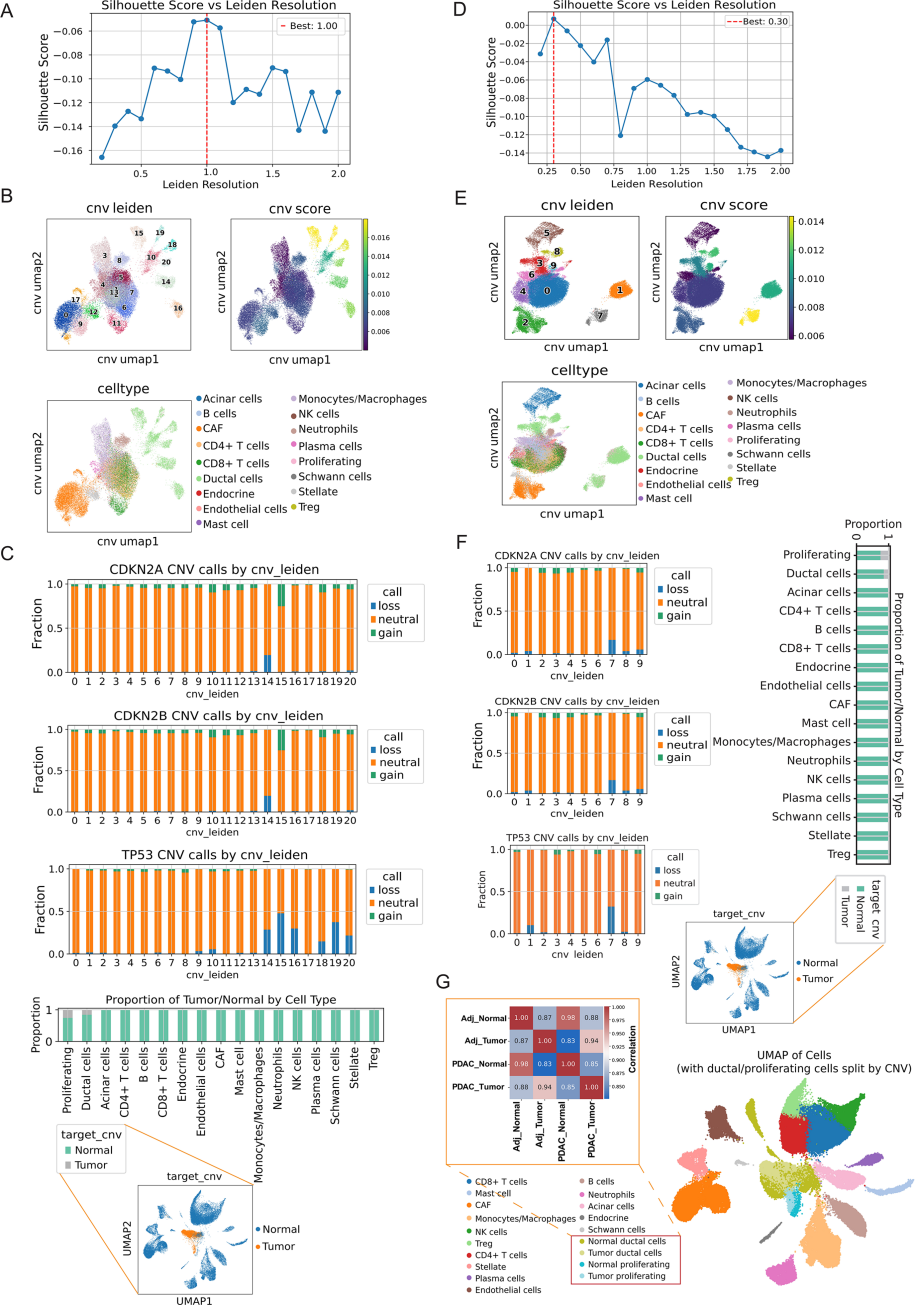
CNV-based clustering delineates malignant ductal and proliferating cells. **(A)** Silhouette scores for CNV-based Leiden clustering in PDAC samples, with the optimal resolution indicated by the red dashed line (best resolution = 1.0). **(B)** UMAP of the CNV embedding in PDAC samples colored by CNV Leiden clusters, inferred CNV score, and annotated cell types. **(C)** Stacked bar plots showing the fractions of cells with CNV loss, neutral, or gain for CDKN2A, CDKN2B, and TP53 across CNV clusters, and the proportions of tumor-like versus normal-like cells within each major cell type in PDAC. **(D)** Silhouette scores for CNV-based Leiden clustering restricted to ductal and proliferating cells in adjacent (Adj) samples, with the optimal resolution highlighted (best resolution = 0.30). **(E)** UMAP of this ductal/proliferating subset in Adj samples, colored by CNV Leiden clusters, CNV score, and cell-type annotations. **(F)** CNV call distributions for CDKN2A, CDKN2B, and TP53 across CNV clusters, and the proportions of tumor-like versus normal-like cells within each duct cells type in Adj samples. **(G)** Heatmap summarizing the concordance between CNV-derived tumor/normal labels and sample group (Adj vs PDAC).

To further clearly identify malignant cells, we performed inferCNV analysis. We first optimized Leiden resolutions by maximizing the silhouette score for CNV-derived embeddings in tumor and adjacent samples (best resolutions 1.0 and 0.3, respectively; **Figures 4A, D**). CNV-level UMAPs revealed discrete clusters with high CNV burden that almost exclusively contained ductal and proliferating cells, whereas immune and stromal populations showed near-neutral CNV scores (**Figures 4B, E**). Gene-level CNV calling further demonstrated that losses of CDKN2A, CDKN2B and TP53 were strongly enriched in CNV-high ductal/proliferating clusters, while remaining largely neutral in non-epithelial lineages (**Figures 4C, F**). Consistently, cells harboring these alterations were predominantly derived from PDAC samples rather than adjacent tissue, and reclustering after splitting ductal/proliferating cells by CNV status clearly separated malignant from non-malignant compartments (**Figure 4G**, **Supplementary Figure 2A**).

### 3.4 Enriched and dynamic deubiquitinase activity along the identified malignant trajectory

**Figure 5:**
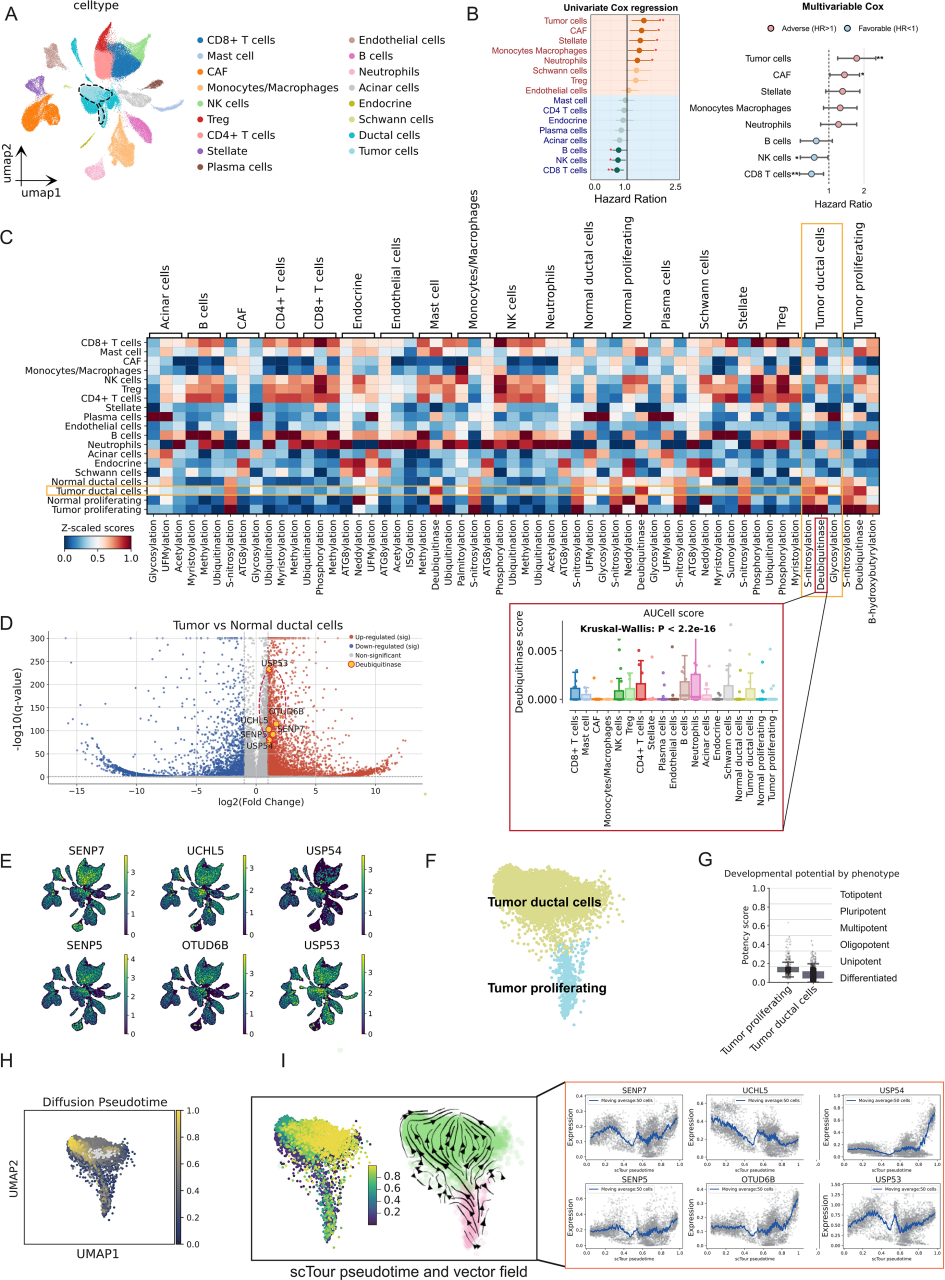
Deubiquitinase activity and expression along the malignant proliferating-ductal axis. **(A)** UMAP of all cells colored by major cell types (ductal tumor cells and ductal tumor proliferating cells are defined as tumor cells, ductal normal cells and ductal normal proliferating cells are defined as normal cells). **(B)** Forest plots showing univariate and multivariable Cox regression of overall survival for each cell type based on their fractional abundance; dots indicate hazard ratios (HRs) and lines 95% CIs, with adverse (HR>1) and favorable (HR<1) cell types highlighted. **(C)** Heatmap of AUCell-based post-translational modification (PTM) signature scores across cell types, with a boxplot of deubiquitinase activity scores showing significant differences among cell types (Kruskal–Wallis p < 2.2×10^−16^). **(D)** Volcano plot of differentially expressed genes between tumor and normal ductal cells; deubiquitinase genes are highlighted. **(E)** UMAP feature plots displaying the expression of representative deubiquitinase genes (SENP7, UCHL5, USP54, SENP5, OTUD6B, USP53). **(F)** UMAP of tumor cells highlighting tumor ductal versus tumor proliferating populations. **(G)** Boxplot of cytotrace2-derived developmental potency scores comparing tumor proliferating and tumor ductal cells. **(H)** Diffusion pseudotime (DPT) embedding. **(I)** scTour pseudotime embedding and vector field (left) and corresponding smoothed expression profiles of the same deubiquitinase genes along the DPT axis (right).

The single-cell atlas confirmed 17 major cell populations, with tumor ductal and tumor proliferating cells formed a continuous malignant continuum (**Figure 5A**). To evaluate their clinical relevance, we used BayesPrism-derived cell fractions in TCGA. Univariable Cox regression revealed that higher abundances of tumor cells, CAFs, stellate cells, monocytes/macrophages and neutrophils were significantly associated with worse overall survival. In addition, multivariate Cox regression analysis further confirmed that tumor cells and CAFs remained independent predictors of poor prognosis (**Figure 5B**). Subsequently, we conducted an in-depth analysis of the molecular characteristics of this malignant continuum. Single-cell PTM activity scoring based on AUCell revealed that the DUB pathway was significantly activated in the malignant cell population, particularly prominent in tumor ductal cells (**Figure 5C**). By comparing the single-cell gene expression profiles of malignant and normal ductal cells, we identified multiple upregulated DUB genes, including *SENP7*, *UCHL5*, *USP54*, *SENP5*, *OTUD6B*, and *USP53* (**Figure 5D**; **Supplementary Table 5**). These genes were not only highly expressed in malignant cells but also predominantly concentrated in the malignant region on the single-cell UMAP plot (**Figure 5E**).

We focused on tumor proliferative and tumor ductal cell populations to explore the dynamic behavior of these cells during malignant advancement (**Figure 5F**). Developmental potential analysis revealed that tumor proliferative cells possess enhanced stem-like characteristics relative to tumor ductal cells, supporting a differentiation trajectory from a proliferative state toward a ductal-like phenotype (**Figure 5G**; **Supplementary Table 6**). We then ordered the cells along this axis using diffusion and scTour pseudotime analyses (**Figures 5H, I**). Pseudotime analysis with scTour revealed distinct dynamic expression patterns among the DUB genes: *USP54*, *OTUD6B*, and *SENP5* were significantly upregulated during late pseudotime, while others showed transient early peaks, biphasic expression, or decreasing trends.

### 3.5 USP54 is identified as a key deubiquitinase in PDAC malignant progression

**Figure 6:**
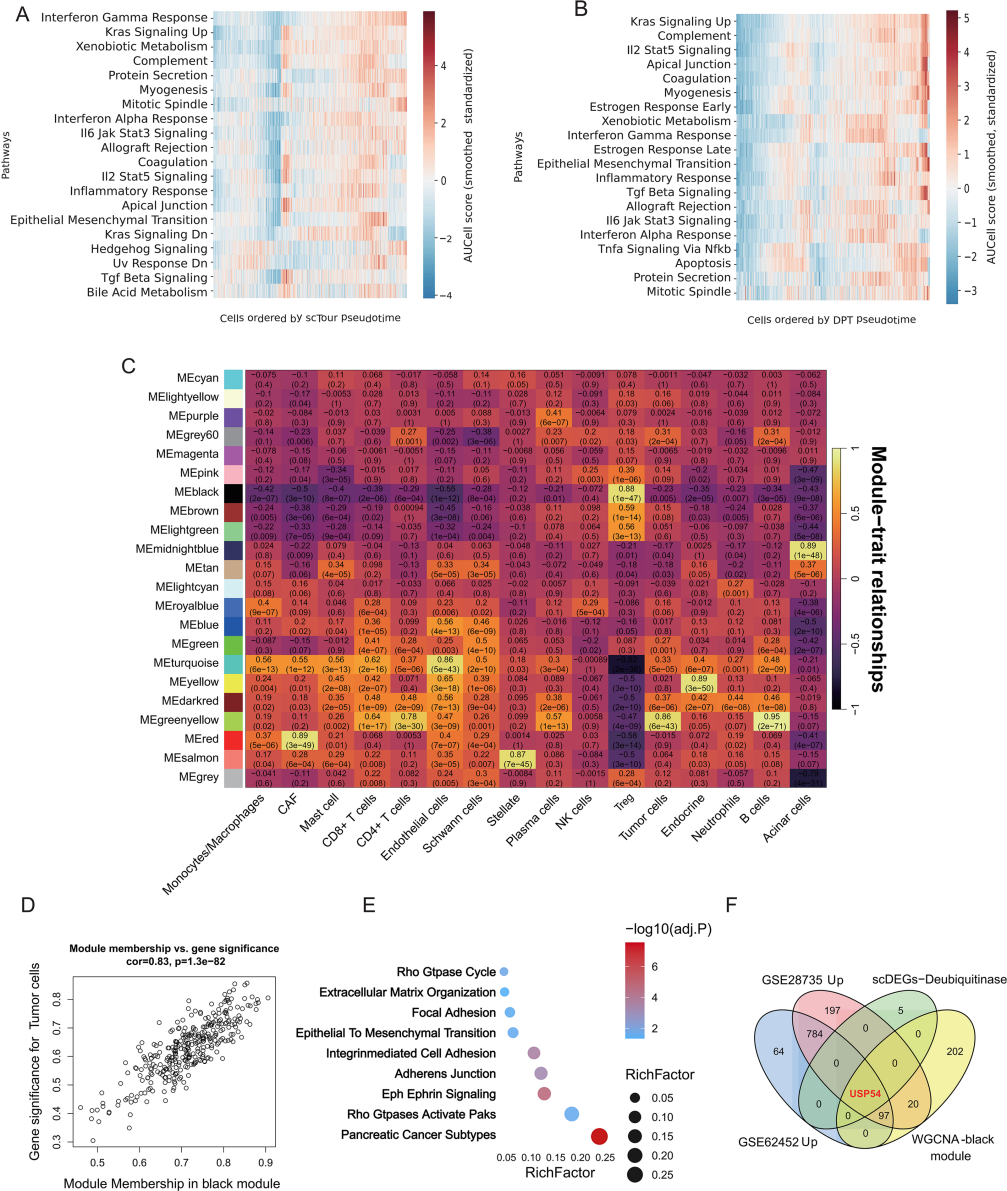
USP54 is identified as a key deubiquitinase in PDAC malignant progression. **(A)** Hallmark pathway AUCell scores along the scTour-derived pseudotime from tumor proliferating to tumor ductal cells. Columns represent single cells ordered by pseudotime; rows are pathways, and colors show Z-scored AUCell values. **(B)** Hallmark pathway AUCell scores along the diffusion pseudotime (DPT) from tumor proliferating to tumor ductal cells. Columns represent single cells ordered by pseudotime; rows are pathways, and colors show Z-scored AUCell values. **(C)** Heatmap of correlations between WGCNA module eigengenes and traits, including tumor cell fractions. Numbers in each tile indicate Pearson correlation coefficients and associated P-values. **(D)** Scatter plot of gene module membership (kME) versus gene significance for the black module, showing a strong positive association between membership in this module. **(E)** Dot plot of KEGG pathways enriched among black-module genes, with dot size indicating gene ratio and color indicating –log10(adjust value). **(F)** Venn diagrams showing overlap between black-module genes and up-regulated genes from independent bulk datasets (GSE28735 and GSE62452) and tumor ductal versus normal ductal cells in scRNA-seq.

To characterise the molecular programs involved in malignant progression, we first examined the pathway activity along the pseudotime trajectory from tumor proliferating to ductal cells. Both scTour and diffusion pseudotime showed an orchestrated upregulation of various inflammatory and oncogenic signaling pathways (e.g., IFN-γ response, KRAS signaling, complement and coagulation, xenobiotic metabolism and TNF signaling) (**Figures 6A, B**). To systematically detect co-expression modules related to tumor cell proportion, we applied weighted gene coexpression network analysis (WGCNA), which detected one major module (“black”) module highly correlated with tumor cell proportion (**Supplementary Table 7**). A high correlation between module membership and gene significance suggested an internally consistent module (**Figures 6C, D**, **Supplementary Figures 3A–D**). Functional enrichment analysis also validated it was related to some biological processes including epithelium mesenchyme transition, RhoGTPase signaling, Eph-ephrin signaling, and pancreatic cancer subtypes (**Figure 6E**). Notably, in order to obtain the strongest core genes, we intersected together 4 gene-sets: black-module genes, DUBs found by sc-differential expression analyses and those genes which were up-regulated in 2 independent PDAC cohorts. This multiple filtering approach found that only one gene was shared among all of these four groups, namely *USP54* (**Figure 6F**), representing a stepwise integration of module significance, cell-type specificity, and cross-cohort consistency to prioritize core drivers, and is therefore likely an important player during PDAC progression.

### 3.6 Evidence supports a putative KLF5-USP54 regulatory axis potentially influenced by the immune microenvironment

**Figure 7:**
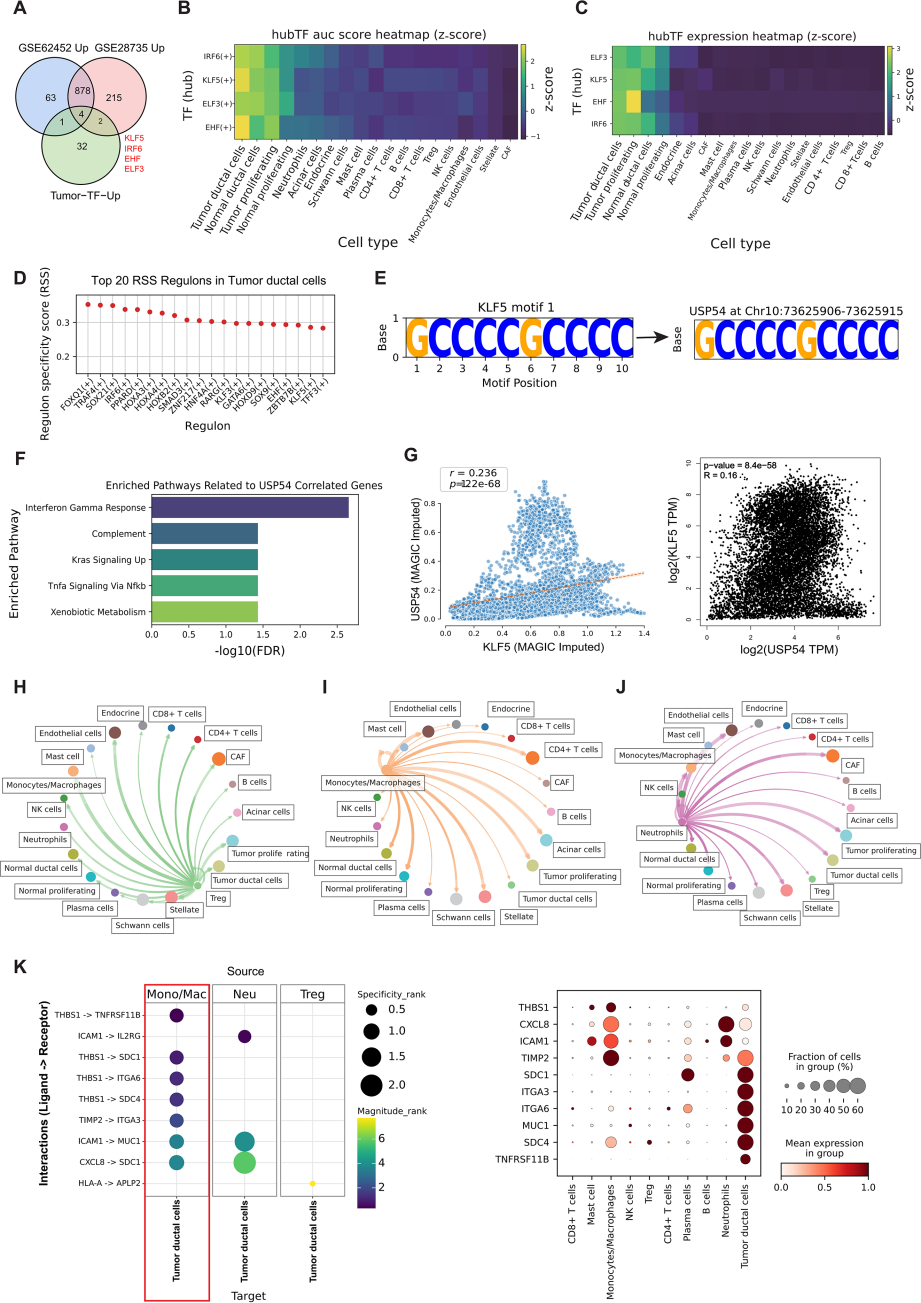
The KLF5-USP54 axis may link tumor ductal cell malignant progression to the immune microenvironment. **(A)** Venn diagrams showing overlap between up-regulated genes from independent bulk datasets (GSE28735 and GSE62452) and up-regulated transcription factors (TFs) from tumor ductal versus normal ductal cells in scRNA-seq. **(B, C)** Heatmaps of hub TFs regulon activity (AUCell Z-score) and TF expression (Z-score) across major cell types. **(D)** Ranked regulon specificity scores (RSS) for tumor ductal cells. **(E)** Schematic of a representative KLF5 binding motif and a FIMO-identified KLF5 motif occurrence in the USP54 promoter region. **(F)** Hallmark pathways enriched among USP54-correlated genes in single cell hypervariable genes. **(G)** Positive correlations between KLF5 and USP54 expression at the single-cell level (MAGIC-imputed tumor ductal cells, upper panel) and across TCGA pan-cancer bulk RNA-seq samples (lower panel). **(H–J)** Strength of ligand–receptor–mediated communication between Treg cells, monocytes/macrophages, neutrophils, and the other cell populations. **(K)** Left panel: LIANA-derived ligand–receptor dot plot showing THBS1–integrin interactions from monocytes/macrophages (Mono/Mac), neutrophils (Neu), and Treg cells to tumor ductal cells (dot size: specificity rank; color: magnitude rank). Right panel: Bubble heatmap depicting the ligand-receptor expression from Mono/Mac to tumor ductal cells across whole cell types.

Building on the identification of USP54, we next explored potential upstream regulatory factors associated with its expression. SCENIC analysis showed that several transcription factors, including KLF5, EHF, ELF3, and IRF6, displayed increased regulon activity and expression in tumor ductal cells (**Figure 7A**). Among these candidates, KLF5 exhibited the highest regulon specificity score (RSS) and was therefore prioritized for further investigation (**Figures 7B–D**; **Supplementary Tables 8–9**). Motif scanning of transcription factor binding sites identified a high-confidence KLF5 binding motif within the USP54 promoter region (**Figure 7E**; **Supplementary Figure 4**), suggesting a potential regulatory link. In addition, single-cell hypervariable genes co-expressed with USP54 were significantly enriched in inflammatory and oncogenic signaling pathways, including IFN-γ response, complement, KRAS signaling, TNF signaling, and xenobiotic metabolism (**Figure 7F**). These pathway enrichments are consistent with the activation patterns observed along the malignant trajectory (**Figures 6A, B**). Furthermore, both at the single-cell level (after MAGIC imputation) and across pan-cancer datasets, KLF5 and USP54 expression showed a strong positive correlation (**Figure 7G**). Taken together, these findings support an association between KLF5 activity and USP54 expression and are consistent with a putative role for KLF5 in the transcriptional regulation of USP54 and its downstream programs; however, this relationship remains correlative and requires further experimental validation. Cell-cell communication analysis revealed strong interactions between monocytes/macrophages, neutrophils, Treg cells and tumor ductal cells (**Figures 7H–J**; **Supplementary Figures 2B–N**). Integrating with transcriptional differential expression data indicated that monocytes/macrophages contributed the largest set of differentially expressed ligand–receptor pairs, suggesting particularly intense cross-talk between monocytes/macrophages and tumor ductal cells (**Figure 7K**; **Supplementary Figure 2O**; **Supplementary Table 10**). Notably, the THBS1-integrin axis emerged as a key mediator. Given previous reports that ITGA6 co-expresses with KLF5 (51), these findings imply a potential model whereby immune-derived signals (e.g., THBS1) via integrins might influence the KLF5-positive tumor cell compartment.

### 3.7 Spatial mapping identifies architecturally distinct tumor niches in PDAC

**Figure 8:**
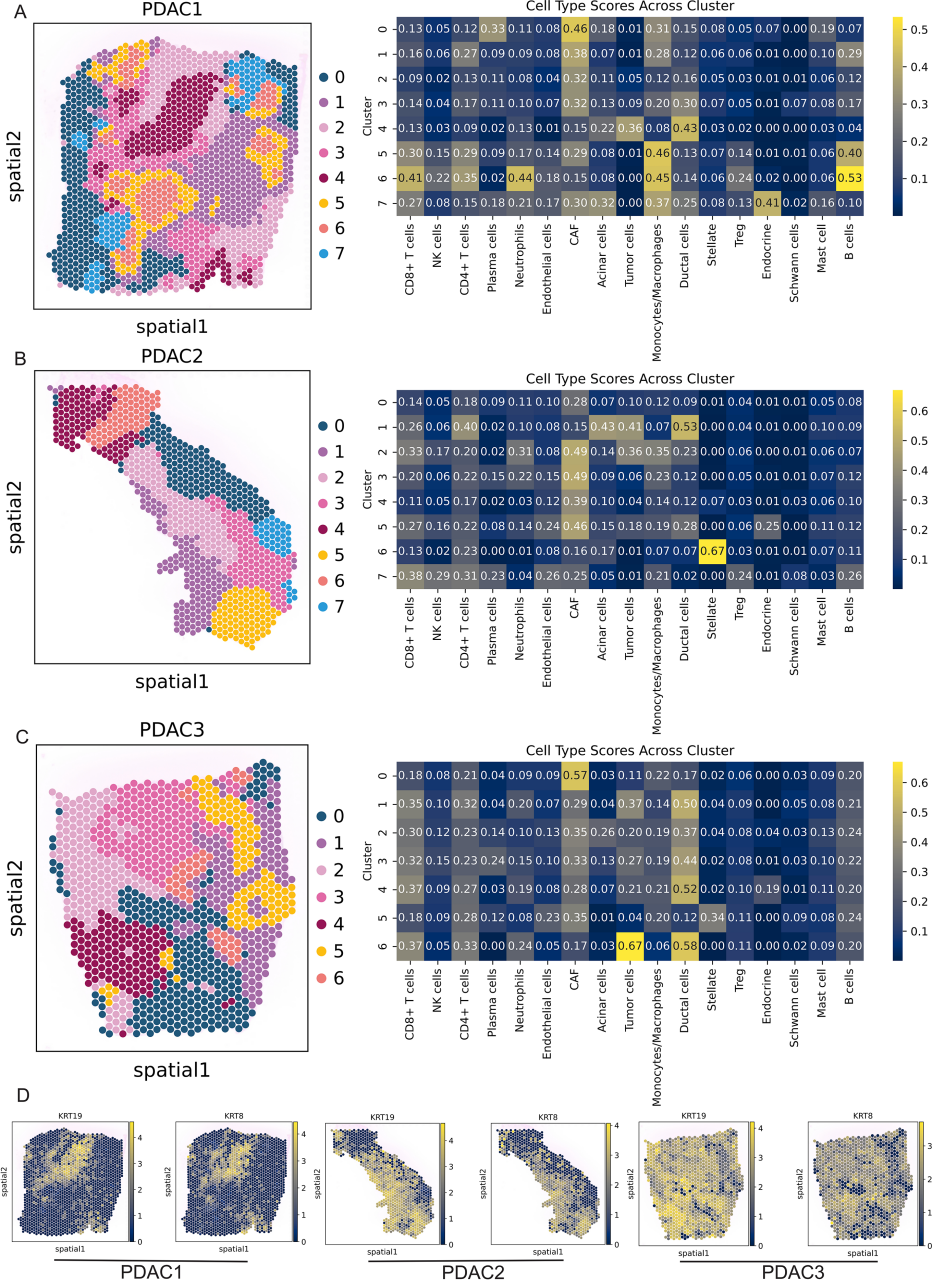
Spatial clustering and cell-type mapping across three PDAC specimens. **(A–C)** Left: Hexagon-binned spatial maps of GraphST-derived clusters for three PDAC Visium samples (PDAC1, PDAC2, PDAC3), with each color indicating a distinct spatial cluster. Right: Heatmaps showing mean cell2location-derived cell-type abundance scores for each cluster, across major immune and stromal populations and tumor compartments. Numbers within tiles indicate scaled abundance values. **(D)** Spatial expression of ductal cells markers KRT19 and KRT8 in PDAC1-3.

To gain some insight into how the PDAC tumor microenvironment is organized spatially, we carried out spatial transcriptomic analyses for three PDAC samples (PDAC1-3), using Visium technology and projected SC defined populations with cell2location, and domains with GraphST, which showed reproducible spatial domains in each sample (**Figures 8A–C**). Spatial deconvolution suggested compartmentalized Micellular composition was regionally predominant: some areas contained a large proportion of malignant ductal/tumor cells, while the remaining were enriched for stromal components (e.g., CAFs and endothelial cells), or immune components (e.g., T-cells, B-cells and monocyte/macrophage) (**Figures 8A–C**; **Supplementary Figure 5**). The expression of the ductal epithelial markers KRT19 and KRT8 confirmed the validity of the spatial map. In both PDAC1 and PDAC2, we observed that these markers localized in space to areas of high inferred ductal/tumor cell abundance, cleanly separating out the tumor core (**Figure 8D**). In PDAC3, however, for example KRT19 and KRT8 had more scattered patterns which reflects the biological heterogeneity of PDAC and thus highlights that spatial analyses need to be done per sample. Combining these marker locations with a cell-type map of the tissue allowed us to determine that cluster 4 is likely the main tumor area within PDAC1 and cluster 1 & 2 as core tumor regions for PDAC2, and Cluster 6 as core tumor region for PDAC3.

### 3.8 Spatial mapping identifies USP54−high, DUB− and KRAS−active tumor niches adjacent to THBS1−enriched immune regions

**Figure 9:**
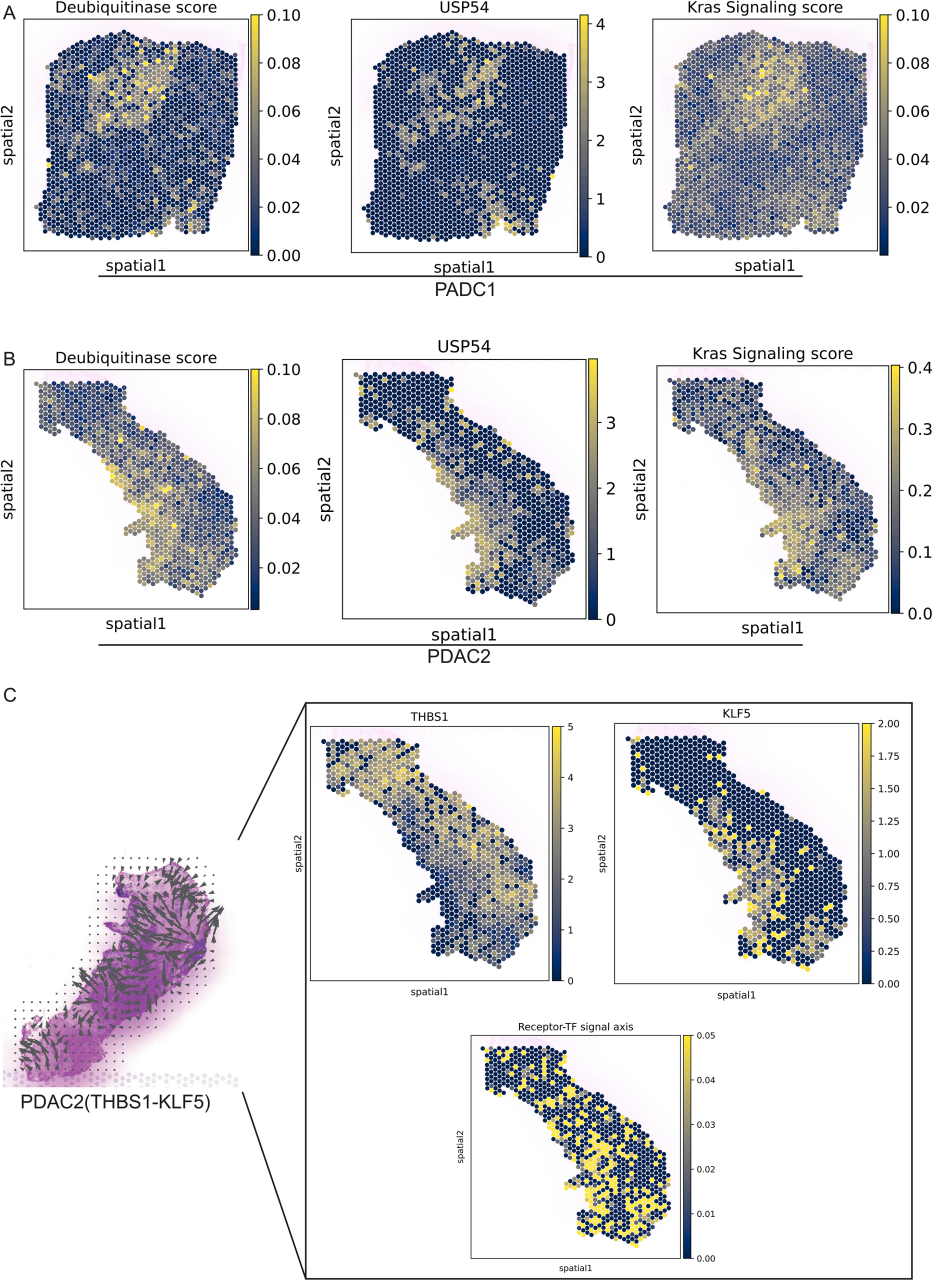
Spatial mapping of USP54, DUB activity, KRAS signaling, THBS1 and KLF5 in PDAC. **(A, B)** Spatial transcriptomic maps of two independent PDAC specimens (PDAC1 and PDAC2) showing the distribution of deubiquitinase activity scores (left; AUCell-derived PTM score), USP54 expression (middle; log-normalized expression), and Kras signaling scores (right; Hallmark KRAS signaling signature). **(C)** In PDAC2, left panel shows the inferred THBS1–KLF5 receptor-to-TF axis overlaid as streamlines on the tissue section. Right panels display spatial expression of THBS1 (top left) and KLF5 (top right), and the composite receptor-to-TF axis score (bottom) derived from THBS1-integrin-KLF5 signaling.

Given that the tumor core regions were clearly delineated in PDAC1 and PDAC2, we restricted spatial validation to these two specimens. Spatial transcriptomics analysis showed that spots with high DUB activity scores were largely confined to tumor areas and closely overlapped with regions of elevated USP54 expression and enhanced KRAS signaling activity (**Figures 9A, B**), indicating functional interrelationships within this specialized niche. Cell type mapping showed that, in PDAC2, the tumor border was infiltrated by monocyte/macrophages, prompting detailed investigation of immune-tumor crosstalk (**Figure 8B**). THBS1 expression was enriched in peritumoral regions abundant with macrophages, while KLF5 expression peaked within tumor compartments (**Figure 9C**). Importantly, a composite receptor-to-TF axis score representing THBS1-integrin-KLF5 signaling partially colocalized with USP54- and KRAS-high domains. Taken together, these data imply that THBS1 signals from tumor-associated monocytes/macrophages converge on KLF5-positive tumor ductal cells to establish a spatially restricted USP54/DUB-KRAS signaling hub in PDAC.

### 3.9 High USP54 expression predicts poor prognosis

**Figure 10:**
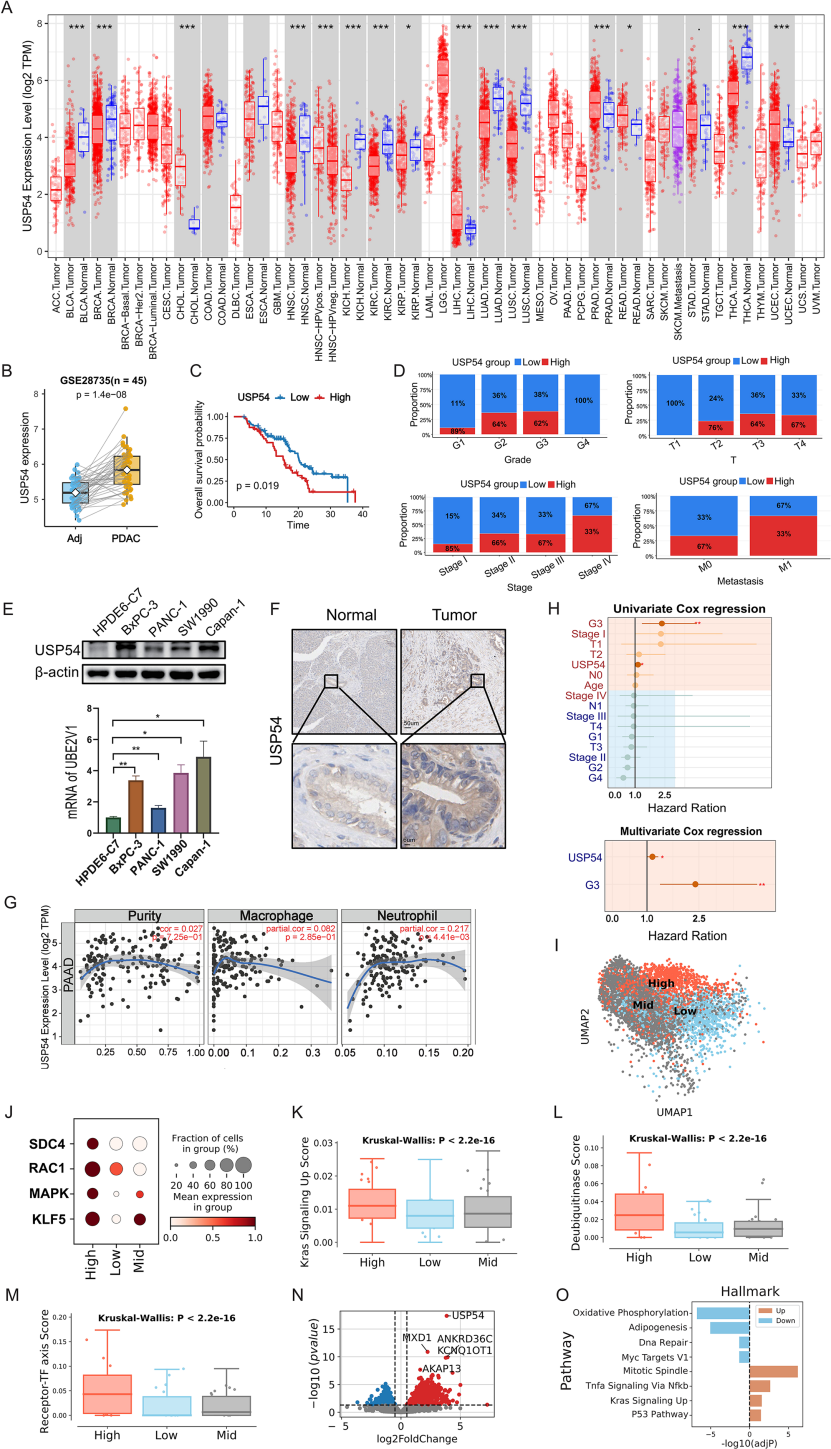
High USP54 expression defines an aggressive subtype with an immune−inflamed microenvironment and poor prognosis. **(A)** USP54 expression across TCGA pan-cancer cohorts, showing log2 (TPM+1) values in tumor (red) versus normal (blue) tissues for each cancer type. **(B)** Paired USP54 expression in two independent pancreatic ductal adenocarcinoma (PDAC) datasets (GSE28735), comparing adjacent (Adj) and tumor (PDAC) tissues from the same patients. **(C)** Kaplan–Meier overall survival curves for patients with high versus low USP54 expression in TCGA-PAAD, stratified by an optimal cutoff; P-value from log-rank test. **(D)** Stacked bar charts show the distribution of clinicopathological features (grade, stage, T and M stage) between USP54 high- and low-expression groups; P-values from χ² tests. **(E)** WB and RT-qPCR analyses of USP54 expression in the normal pancreatic cell line HPDE6-C7 and PDAC cell lines (n=3, one-way ANOVA). **(F)** IHC staining of USP54 in tumor and matched non-tumor tissues from a PDAC patient. **(G)** Associations between USP54 expression and tumor purity or immune infiltration of macrophages and neutrophils; each scatter plot shows log2 (TPM + 1) USP54 expression versus inferred infiltration level, with partial correlation coefficient and P-value indicated. **(H)** Forest plots of univariate and multivariable Cox regression analyses assessing the prognostic impact of USP54 expression on overall survival in TCGA-PAAD; hazard ratios (HRs) and 95% confidence intervals are shown. **(I)** UMAP of PDAC with tumor ductal cells colored by USP54-based stratification (Low, Mid, High). **(J)** Dot plot showing expression of selected receptor-TF axis genes across USP54 Low, Mid, and High tumor ductal groups; dot size indicates the fraction of cells expressing the gene and color represents mean expression. **(K)** KRAS signaling activity score across USP54 Low, Mid, and High tumor ductal groups; P-value from Kruskal–Wallis test. **(L)** Deubiquitinase activity score across USP54 groups in tumor ductal cells; P-value from Kruskal–Wallis test. **(M)** Receptor–TF axis score (THBS1–integrin–KLF5) across USP54 groups in tumor ductal cells; P-value from Kruskal–Wallis test. **(N)** Volcano plot of differentially expressed genes between USP54 High and Low tumor ductal cells; vertical dashed lines mark log2 fold-change thresholds and the horizontal line marks the adjusted P-value cutoff. **(O)** Bar plot of Hallmark pathways significantly enriched among genes up and down-regulated in USP54-High tumor ductal cells. *p < 0.05,***p < 0.001.

To further define the clinical significance of USP54, we first analyzed TCGA pan-cancer cohorts and found differential USP54 expression across multiple tumor types compared to matched normal tissues (**Figure 10A**). Since TCGA-PAAD lacks normal tissue controls, we validated this finding in the GEO dataset (GSE28735), confirming significant USP54 upregulation in PDAC compared to adjacent pancreatic tissue (**Figure 10B**). In the TCGA-PAAD cohort, patients with high USP54 expression had significantly worse overall survival than those with low expression (log-rank *p* = 0.019; **Figure 10C**). The proportion of high USP54 expression exhibited a progressive increase across histological grades, more progressed T stages, TNM stages and metastatic cases (**Figure 10D**). More importantly, both mRNA and protein levels of USP54 were markedly elevated in PDAC cell lines (BxPC-3, PANC-1, SW1990 and Capan-1) relative to the normal human pancreatic duct epithelial cell line HPDE6-C7 (**Figure 10E**). Immunohistochemical (IHC) analysis of paired clinical specimens further confirmed the upregulation of USP54 in PDAC tissues compared to matched non-tumor tissues (**Figure 10F**). Furthermore, higher USP54 expression correlated with increased tumor purity and enhanced infiltration of macrophage and neutrophil (**Figure 10G**), suggesting that USP54-high tumors reside in an immune-inflamed microenvironment. Both univariable and multivariable Cox regression analyses confirmed USP54 as an independent adverse prognostic factor (**Figure 10H**).

At the single-cell level, we stratified malignant ductal and proliferating cells into USP54-low, -mid, and -high states. USP54-high cells formed a distinct cluster in UMAP space (**Figure 10I**) and demonstrated coordinated upregulation of KLF5 (**Figure 10J**). These cells exhibited the highest KRAS signaling activity scores (**Figure 10K**), along with significantly elevated DUB-related and receptor-TF scores (**Figures 10L, M**). Comparative single-cell transcriptomic analysis between USP54-high and -low tumors identified *USP54* and a set of co-upregulated genes (e.g., *AKAP13*, *MXD1*, *ANKRD36C*, *KCNQ1OT1*) (**Figure 10N**). Pathway enrichment analysis of these genes highlighted involvement in mitotic spindle, TNFα/NF-κB and KRAS signaling (**Figure 10O**), helping to define the aggressive molecular phenotype of USP54-high PDAC.

### 3.10 USP54 knockdown inhibits PDAC cell proliferation and metastasis

**Figure 11:**
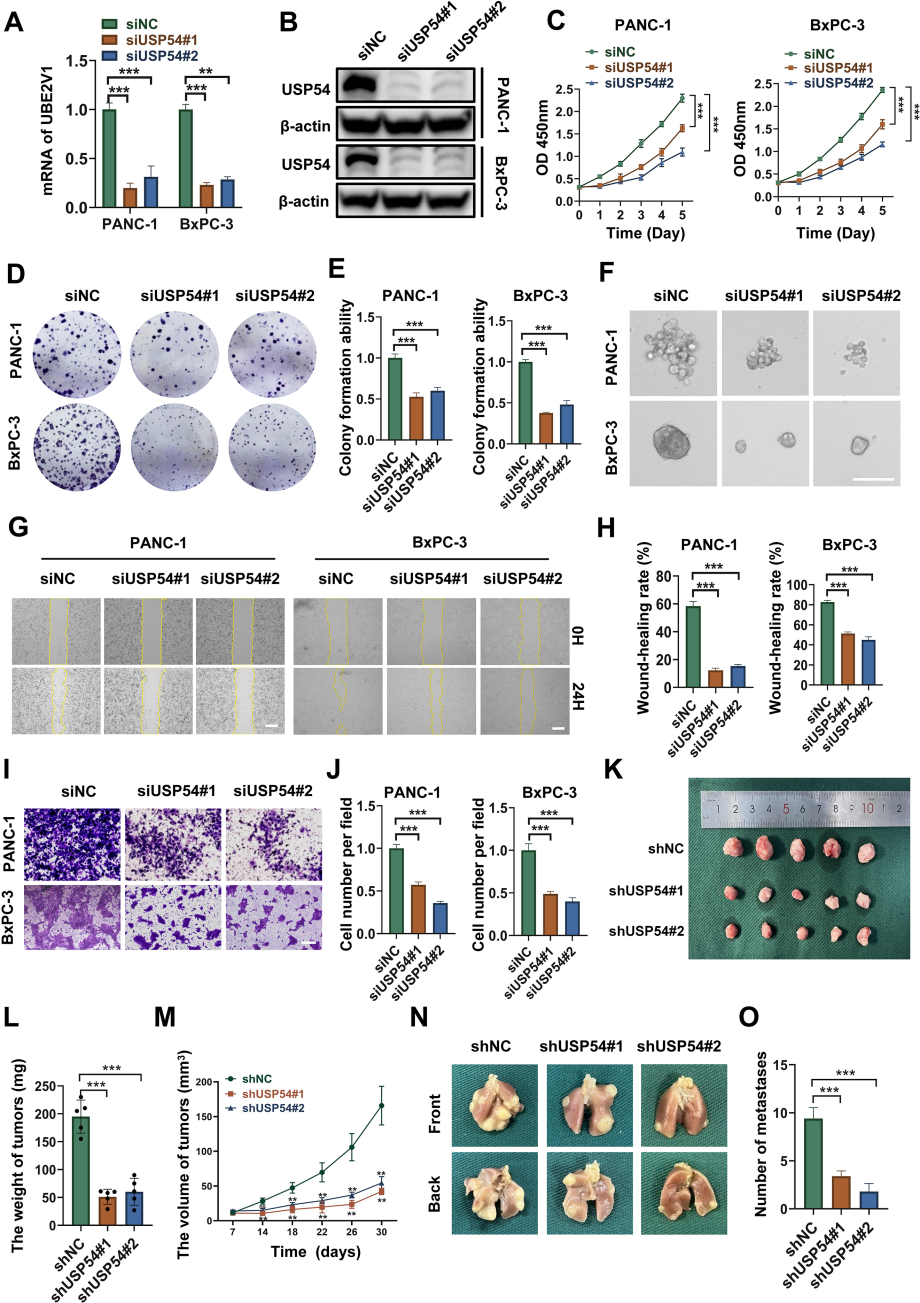
USP54 knockdown impairs proliferation and migration in PDAC cells. **(A, B)** Silencing of USP54 in BxPC-3 and PANC-1 cells was verified by RT-qPCR and western blotting (n=3; one-way ANOVA). **(C–E)** Proliferation was assessed by CCK-8 and colony formation assays, showing significant suppression upon USP54 depletion (n=3; one-way ANOVA). **(F)** Tumor sphere formation was notably reduced following USP54 knockdown. **(G–J)** Cell migration was impaired in both transwell and wound healing assays after USP54 silencing (n=3; one-way ANOVA). **(K)** Representative photographs of excised subcutaneous tumors from nude mice. **(L)** Final tumor weights measured at the endpoint (n=5, one-way ANOVA). **(M)** Tumor growth curves measured over time (n=5, two-way RM-ANOVA). **(N, O)** Lung metastasis assessment in the tail vein injection model (n=3, one-way ANOVA). Scale bars: 200 μm. The sequence (5’-3’) of siUSP54#1: GCUGGAAAGACGAGAGAAATT; The sequence (5’-3’) of siUSP54#2: GAAGCAGGCUCCUAGAAAUTT. *p < 0.05, **p < 0.01, ***p < 0.001.

We performed loss-of-function studies to validate the oncogenic role of USP54 predicted by our multi-omics data. In BxPC-3 and PANC-1 cells, transfection with two distinct siRNAs targeting USP54 achieved effective knockdown, confirmed at both mRNA and protein levels (**Figures 11A, B**). USP54 depletion markedly reduced tumor cell growth, as shown by decreased viability in CCK-8 assays, impaired colony formation, and reduced tumor sphere formation (**Figures 11C–F**). USP54 silencing also significantly suppressed migration in both transwell and wound healing assays (**Figures 11G–J**). To assess its tumor-promoting function *in vivo*, we established subcutaneous xenografts using PANC-1 cells stably transduced with lentivirus expressing either shRNA against USP54 (shUSP54) or a control shRNA (shNC). USP54 knockdown inhibited tumor growth, resulting in smaller tumor volume and weight (**Figures 11K–M**). Consistent with this model, lung metastatic burden was reduced following USP54 deletion in tail vein injection experiments (**Figures 11N, O**). Together, these results demonstrate that USP54 promotes proliferation and metastasis in PDAC cells.

### 3.11 Ectopic USP54 expression enhances tumorigenic properties

**Figure 12:**
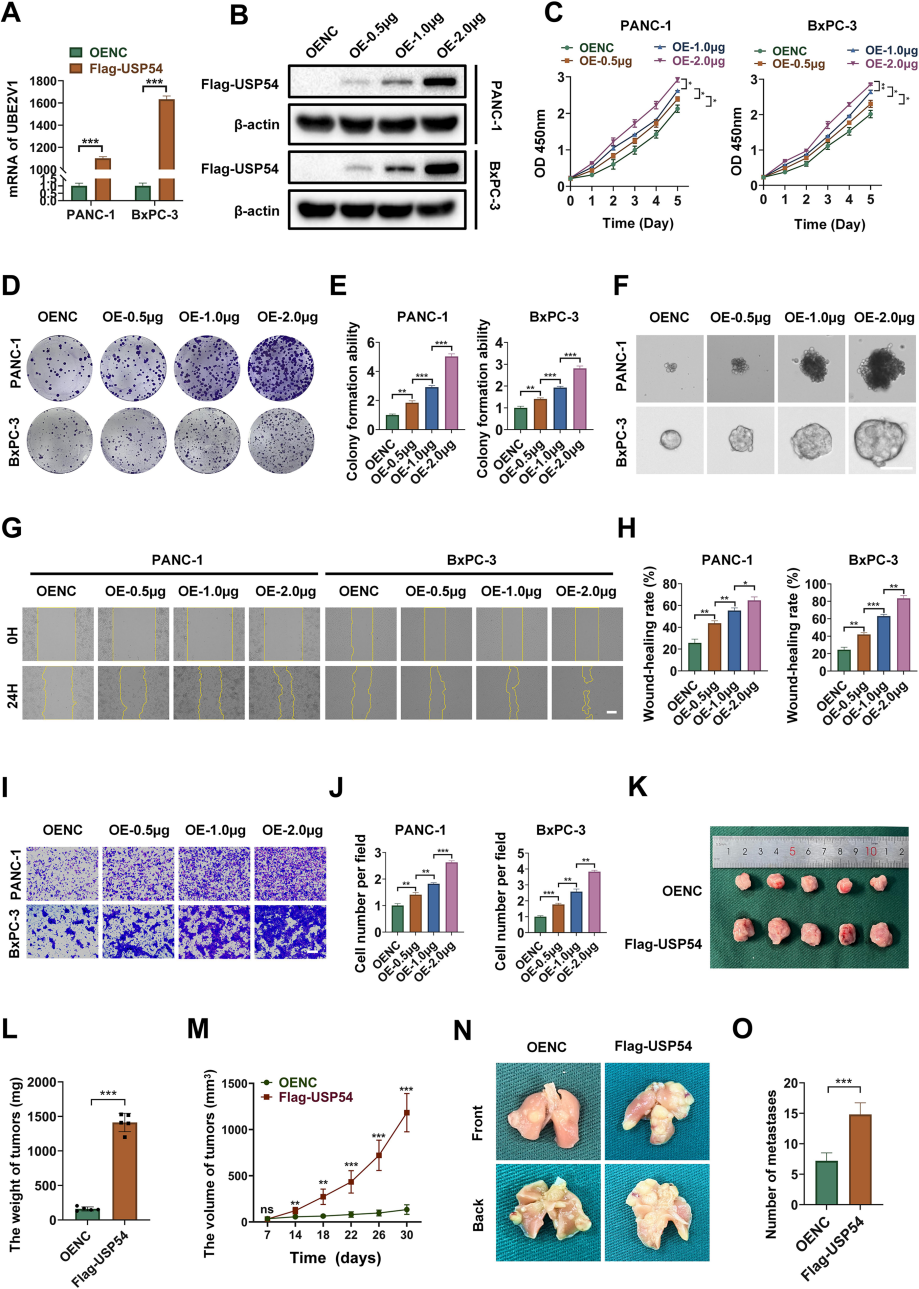
USP54 overexpression promotes the malignant behavior of PDAC cells. **(A, B)** Ectopic expression of USP54 was confirmed via RT-qPCR and western blot analysis. **(C–E)** Overexpression of USP54 promoted cell proliferation, as measured by CCK-8 and colony formation assays (n=3; unpaired Student’s t-test). **(F)** Spheroid formation capacity was strengthened by USP54 upregulation. **(G–J)** Increased cell migration was observed in wound healing and transwell assays under USP54 overexpression (n=3; unpaired Student’s t-test). **(K)** Representative photographs of excised subcutaneous tumors from nude mice. **(L)** Final tumor weights measured at the endpoint (n=5, one-way ANOVA). **(M)** Tumor growth curves measured over time (n=5, two-way RM-ANOVA). **(N, O)** Lung metastasis assessment in the tail vein injection model (n=3, one-way ANOVA). Scale bars: 200 μm. *p < 0.05, **p < 0.01, ***p < 0.001.

We next asked whether USP54 overexpression is sufficient to promote tumorigenic phenotypes. Ectopic expression of USP54 was verified at both the mRNA and protein levels (**Figures 12A, B**). Functionally, USP54 overexpression potently enhanced cellular growth in a dose−dependent manner, as evidenced by a marked increase in cell viability in CCK-8 assays (**Figure 12C**), enhanced colony-forming efficiency (**Figures 12D, E**) and increased sphere-forming capacity (**Figure 12F**). Moreover, USP54 enhanced metastatic potential, as demonstrated by accelerated gap closure in wound healing assays (**Figures 12G, H**) and by a significant increase in traversed cell number in transwell migration assays (**Figures 12I, J**). *In vivo*, subcutaneous implantation of USP54−overexpressing cells resulted in enhanced tumor growth, as indicated by significant increases in both tumor volume and weight compared to control groups (**Figures 12K–M**). In a complementary model, tail vein injection of these cells resulted in more lung metastatic foci (**Figures 12N, O**). Combined with loss-of-function data, the gain-of-function results support a role for USP54 in driving proliferative and metastatic phenotypes in PDAC. The results of this functional study indicate that the high expression of USP54 is directly associated with poor prognosis in patients, suggesting its potential value as a therapeutic target.

## 4 Discussion

In this study, we integrated machine learning, single-cell transcriptomics, and spatial profiling to elucidate key pathways and cellular interactions associated with PDAC aggressiveness. Our analysis converged onto the deubiquitinase USP54 in malignant ductal cells, which correlates with increased deubiquitinase expression and reduced patient survival. We found that USP54 is an indicator for aggressive PDAC whose expression could be predicted to be transcriptionally controlled by the oncogene KLF5, SCENIC and promoter motif scan-based. The proposed regulatory interaction was supported by an observed strong positive correlation in KLF5 and USP54 expression across cells, but also cell-cell communication suggested regulation of this axis through TAM microenvironmental signals. Clinically, USP54 was remarkably upregulated in the PDAC tissues and high expression of USP54 was highly associated with poor prognosis. Functionally, USP54 overexpression promoted PDAC cell proliferation and migration. Taken together, these findings define an isolated PDAC cell state with high levels of post-translational modification activity, for which the role of USP54 has been implicated and therefore this protein may act as either a prognostic marker or therapeutic target.

We first mapped the short-term survival risk of PDAC by ML-based pathway analysis: among many GOs, PTM pathways, especially those related to deubiquitination were found to be most relevantly associated with poor outcome. A PTM-related genes signature-based FSVM model showed optimal performance (C-index up to 0.74 for training set and 0.60 for independent test sets), then others further support that these pathways are relevant for PDAC prognosis. Interestingly, DUB related gene sets (e.g. signatures of deubiquitinases) had a positive contribution on the risk for mortality, whereas pathways restraining cell proliferation were protective. This suggests an environment in which dysregulated protein homeostasis may allow cancer cells to bypass stress and proliferation checkpoints, in line with previous findings indicating that deubiquitinases are often aberrantly expressed in cancer (13). This led us to identify aberrant DUB function as a key driver of PDAC progression.

To complement and extend our pathway analyses, we utilized scRNA-seq data from PDAC tumors and adjacent normal tissues to construct a comprehensive cellular atlas. All major cell populations including malignant ductal cells, proliferating tumor cells, CAFs, stellate cells, and immune subsets (T cells, B cells, NK cells, monocytes/macrophages, neutrophils, among others) were captured and their identities confirmed using canonical marker genes and CNV profiling. Importantly, malignant compartments were clearly delineated based on widespread CNV alterations, including recurrent deletions of tumor suppressor genes *CDKN2A/B* and *TP53*, in contrast to benign ductal cells, which exhibited stable, diploid CNV profiles. This enabled a purified characterization of tumor cells for downstream analyses. We further demonstrated that increased proportions of tumor cells and CAFs in bulk tumor samples are independently associated with reduced OS, consistent with the well-established impact of tumor cellularity and desmoplastic stroma on PDAC prognosis (52). In particular, focusing on the malignant ductal lineage, trajectory analysis revealed a progressive transition from a proliferative, stem-like state toward a more differentiated ductal state. Notably, this transition was accompanied by marked changes in the expression levels of several DUBs, including SENP7, UCHL5, USP54, SENP5, OTUD6B, and USP53. scTour analysis revealed that the expression of several DUBs (such as SENP7, UCHL5, USP54) showed increasing expression toward late pseudotime, with peak expression observed in the most advanced differentiated tumor ductal cells. The enrichment of DUB activity in malignant cells observed here dovetails with these findings and nominates DUBs as potential vulnerabilities in PDAC.

Moreover, *USP54* was identified as the only DUB gene intersected from four gene sets: the key module genes from WGCNA, differentially expressed DUBs from single−cell analysis, and genes consistently upregulated in two independent PDAC cohorts. The functional role of USP54 appears to be context−dependent across cancer types. For instance, in lung adenocarcinoma (LUAD), USP54 is downregulated compared to normal tissue and has been reported to suppress tumor cell proliferation and aerobic glycolysis through a p53−dependent reduction in GLUT1 expression (12), which aligns with its lower expression in LUAD as shown in our pan−cancer analysis (**Figure 10A**). In contrast, USP54 interacts with CEP120 to promote centrosome amplification and tumor progression by stabilizing PLK4 in gastric cancer (53), which aligns with our observation of its high expression in stomach adenocarcinoma (**Figure 10A**). Consistent with observed in our study (**Figure 10A**), USP54 showed high expression in stomach adenocarcinoma (90% of GC) compared with normal tissue. Although USP54 genetic alterations in pancreatic cancer have been previously reported based on public database analyses, functional validation in PDAC has not been performed (13). Here, we demonstrate that USP54 is overexpressed in PDAC tissues relative to matched normal samples, and high USP54 expression correlates with worse overall survival. Functional experiments confirmed that USP54 acts as a pro-tumorigenic factor in PDAC. Overexpression of USP54 enhanced cell proliferation, migration, and sphere-forming capacity, whereas its knockdown suppressed these malignant phenotypes. Collectively, these findings indicate that USP54 promotes PDAC progression and may therefore represent a promising therapeutic target.

Through KLF5 and co-regulated transcription factors (EHF, ELF3, IRF6) showed higher activity in tumor ductal cells than in normal counterparts, with KLF5 displaying the highest regulon specificity score. KLF5, a Krüppel-like factor, promotes cell proliferation and cell-cycle progression in multiple cancers, including PDAC (54, 55). Prior studies have identified KLF5 as an important prognostic biomarker in pancreatic cancer that promotes G1/S cell-cycle transition and tumor growth (56). Consistent with its reported oncogenic role, our data associate KLF5 with increased USP54 expression in PDAC. We identified a high-confidence KLF5 binding motif within the USP54 promoter and observed a strong positive correlation between KLF5 expression/activity and USP54 levels at the single-cell level. Together, these findings are consistent with a potential regulatory relationship in which KLF5 may be linked to USP54 upregulation as part of a broader pro-tumor transcriptional program. However, these observations are correlative and do not establish direct transcriptional regulation, which will require further experimental validation.

Several lines of evidence suggest a connection between the KLF5-USP54 axis and the immune microenvironment. Analysis revealed that PDAC cells with high *USP54* expression were enriched in gene programs related to interferon-γ response, TNFα/NF-κB signaling, and other inflammation/stress pathways. Consistently, in bulk tumor samples, elevated USP54 also showed increased infiltration of immune cells, particularly macrophage and neutrophils, suggesting a more inflamed microenvironment that may favor tumor progression (52). Through cell-cell communication and ligand–receptor analysis, we identified that monocyte/macrophage-derived thrombospondin-1 (THBS1) as a candidate mediator linking immune cells to the KLF5-USP54 axis in tumor cells. THBS1, a matricellular protein, can bind tumor-cell integrins and other receptors on cancer cells to transduce pro-tumor signals. We observed that THBS1 was enriched at the tumor borders heavily infiltrated by macrophages, while its putative target receptor–TF pathway was most active within the adjacent tumor cell clusters. Notably, USP54-high and DUB-active regions spatially overlapped with areas of elevated THBS1-integrin-KLF5 and KRAS signaling. This spatial alignment supports a potential model in which macrophage-derived THBS1 activates integrin-mediated signaling in neighboring tumor cells, which may reinforce KLF5-dependent transcription of *USP54* and other malignancy-associated genes, potentially creating a feed-forward loop that enhances local tumor aggressiveness. This model is consistent with emerging roles of THBS1 in other malignancies. In colorectal cancer, THBS1-expressing monocyte-like cells foster an immunosuppressive and pro-metastatic niche (57). In that context, THBS1 targeting improved responses to immune checkpoint inhibition, identifying it as a relevant stromal immune modulator. Similarly, our findings raise the possibility that in PDAC, macrophage-derived THBS1 promotes integrin-KLF5 signaling in cancer cells, thereby contributing to a DUB-high, inflammatory phenotype. This crosstalk may help explain the poor outcomes in USP54-high tumors, which exhibit high immune infiltration yet display an immune environment polarized toward tumor-promoting inflammation rather than antitumor immunity.

Despite these findings on the role of USP54 in PDAC, we acknowledge several limitations of our study. First, both the scRNA-seq and spatial transcriptomic analyses were derived from a limited number of PDAC cases and tissue sections, and the spatial observations should therefore be interpreted with caution. While these data provide supportive mechanistic insights into the spatial organization of the KLF5–USP54 axis and DUB-high tumor subsets, their generalizability requires validation in larger, independent cohorts with expanded spatial sampling. Second, although integrative analyses suggest a regulatory relationship between KLF5 and USP54, additional experiments are needed to determine whether KLF5 directly regulates USP54 transcription, such as chromatin immunoprecipitation and luciferase reporter assays. Third, cell–cell communication analyses based on ligand–receptor inference are inherently probabilistic and subject to methodological noise. Although we applied multiple complementary communication analysis frameworks and focused on interactions consistently identified across methods to improve robustness, these results remain inferential and require experimental validation. Fourth, due to substantial missing clinical data in the TCGA cohort, several established prognostic factors could not be included in the multivariable analyses, and further validation with more complete clinical annotation is needed. Fifth, pharmacological inhibitors targeting USP54 are currently unavailable, and future translational efforts will require the development of such agents and systematic evaluation of their therapeutic potential in clinically relevant PDAC models. In conclusion, our study characterizes PDAC tumor cell states and their interactions with the tumor microenvironment, and identifies a KLF5-associated USP54 expression program that is associated with malignant features. These findings help bridge genomic risk signatures with potential biological mechanisms. Further investigation could contribute to ongoing efforts to develop novel therapeutic strategies for PDAC.

## Data Availability

The datasets presented in this study can be found in online repositories. The names of the repository/repositories and accession number(s) can be found in the article/**Supplementary Material**.

## Supplementary material

Supplementary Figure 1 Time-specific discrimination performance of four survival models across datasets.

Supplementary Figure 2 Cell-cell communication landscape between immune subsets and tumor ductal cells.

Supplementary Figure 3 WGCNA modules associated with tumor ductal cell abundance.

Supplementary Figure 4 Sequence logos of KLF5, EHF, and ELF3 binding motifs in the USP54 promoter.

Supplementary Figure 5 Spatial distribution of inferred cell‐type abundances in three PDAC specimens.

Supplementary Table 1 Post-translational modification gene set.

Supplementary Table 2 Target sequences for siRNA and shRNA.

Supplementary Table 3 Primer sequences for RT-qPCR in this study.

Supplementary Table 4 List of antibodies used in the study.

Supplementary Table 5 Single-cell differential analysis results.

Supplementary Table 6 Analysis results of CytoTRACE2.

Supplementary Table 7 WGCNA core module genes.

Supplementary Table 8 Results of transcription factor activity analysis from pyscenic.

Supplementary Table 9 Results of differential analysis of transcription factor activity among cell types.

Supplementary Table 10 Cell communication results based on LANA+.
