# Supplementary material for: Multi-omics and experimental validation identify USP54 as a prognostic deubiquitinase promoting pancreatic ductal adenocarcinoma progression within the immune microenvironment
Source: Front Immunol. 2026 Mar 18;17:1791707. doi: 10.3389/fimmu.2026.1791707 (PMC13038871; doi:10.3389/fimmu.2026.1791707)
Supplement: Supplementary Figure 1 — Time-specific discrimination performance of four survival models across datasets. [file DataSheet1.docx]

Supplementary Material


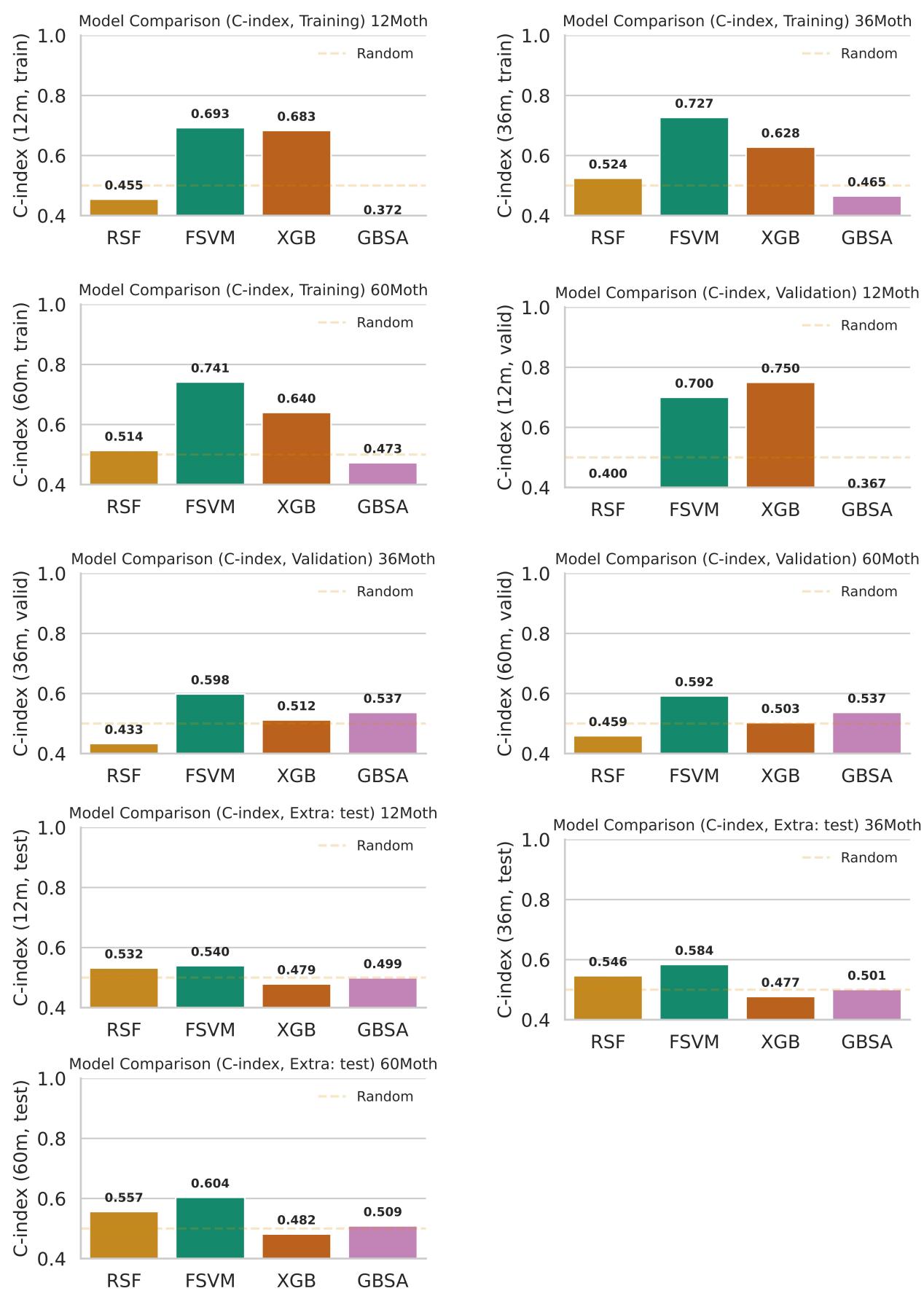


**Supplementary Figure 1.** Time-specific discrimination performance of four survival models across datasets. Bar plots of C-index for Random Survival Forest (RSF), Fast Kernel Survival SVM (FSVM), eXtreme Gradient Boosting (XGB), and Gradient Boosted Survival Analysis (GBSA) in the training cohort at 12, 36 and 60 months, respectively.


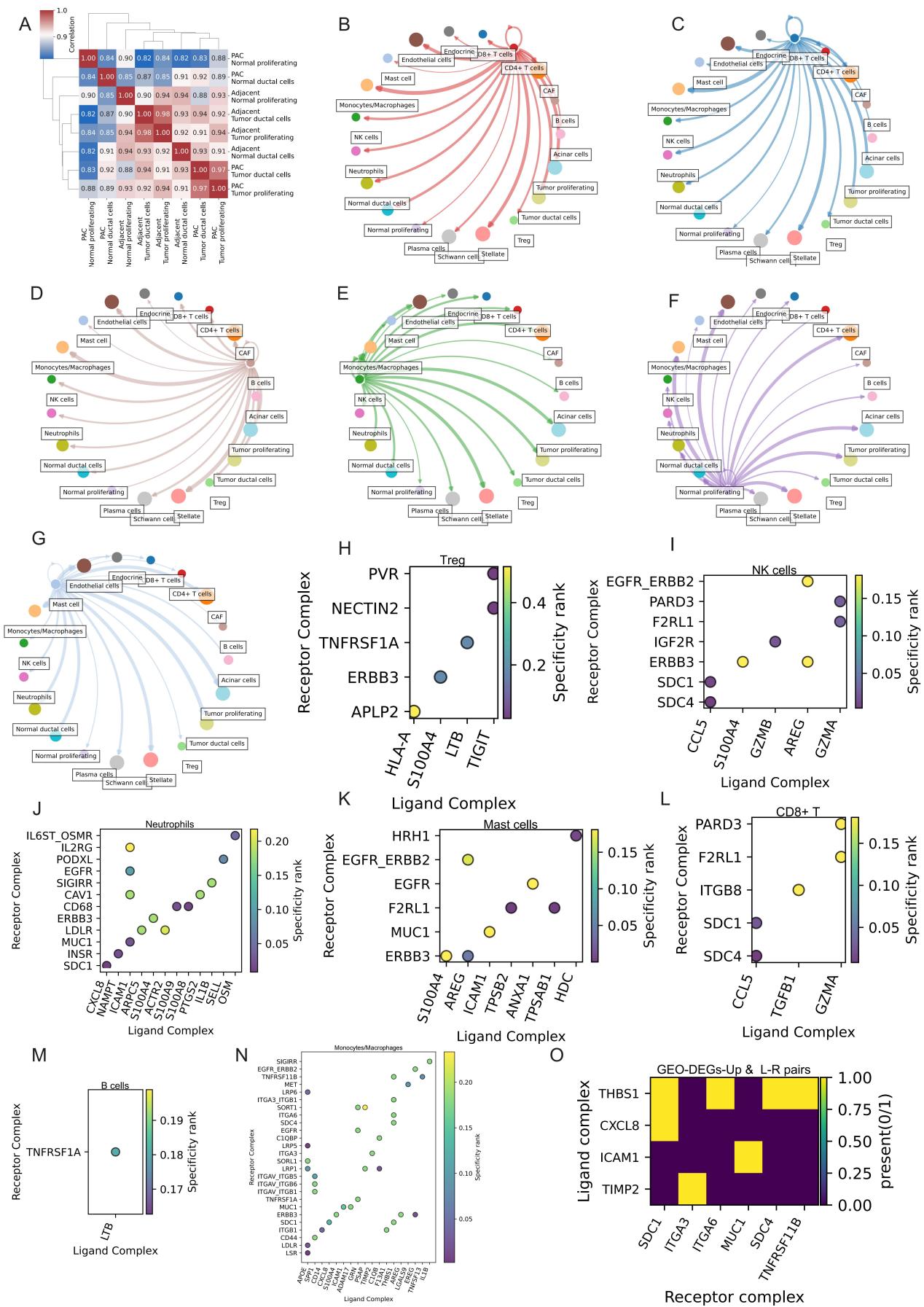


**Supplementary Figure 2.** Cell-cell communication landscape between immune subsets and tumor ductal cells. **(A)** Correlation heatmap between major cell types. **(B, C, D, F, G)** Circle plots summarizing intercellular communication networks for selected immune subsets, with arrows pointing from sender to receiver cell types. Edge thickness reflects overall interaction strength. **(H, I, M)** Bubble plots displaying the significantly regulated ligand–receptor complexes mediating interactions from Treg cells **(H)**, NK cells **(I)**, neutrophils **(J)**, mast cells **(K)**, CD8⁺ T cells **(L)**, B cells **(M)** and monocytes/macrophages **(N)** to tumor ductal cells. Bubble size represents the specificity rank of each ligand–receptor pair, and color encodes the magnitude rank of the interaction score. **(O)** Binary heatmap indicating whether ligand and receptor components of the highlighted pairs are up-regulated in bulk GEO differential-expression datasets (yellow, present; dark, absent).


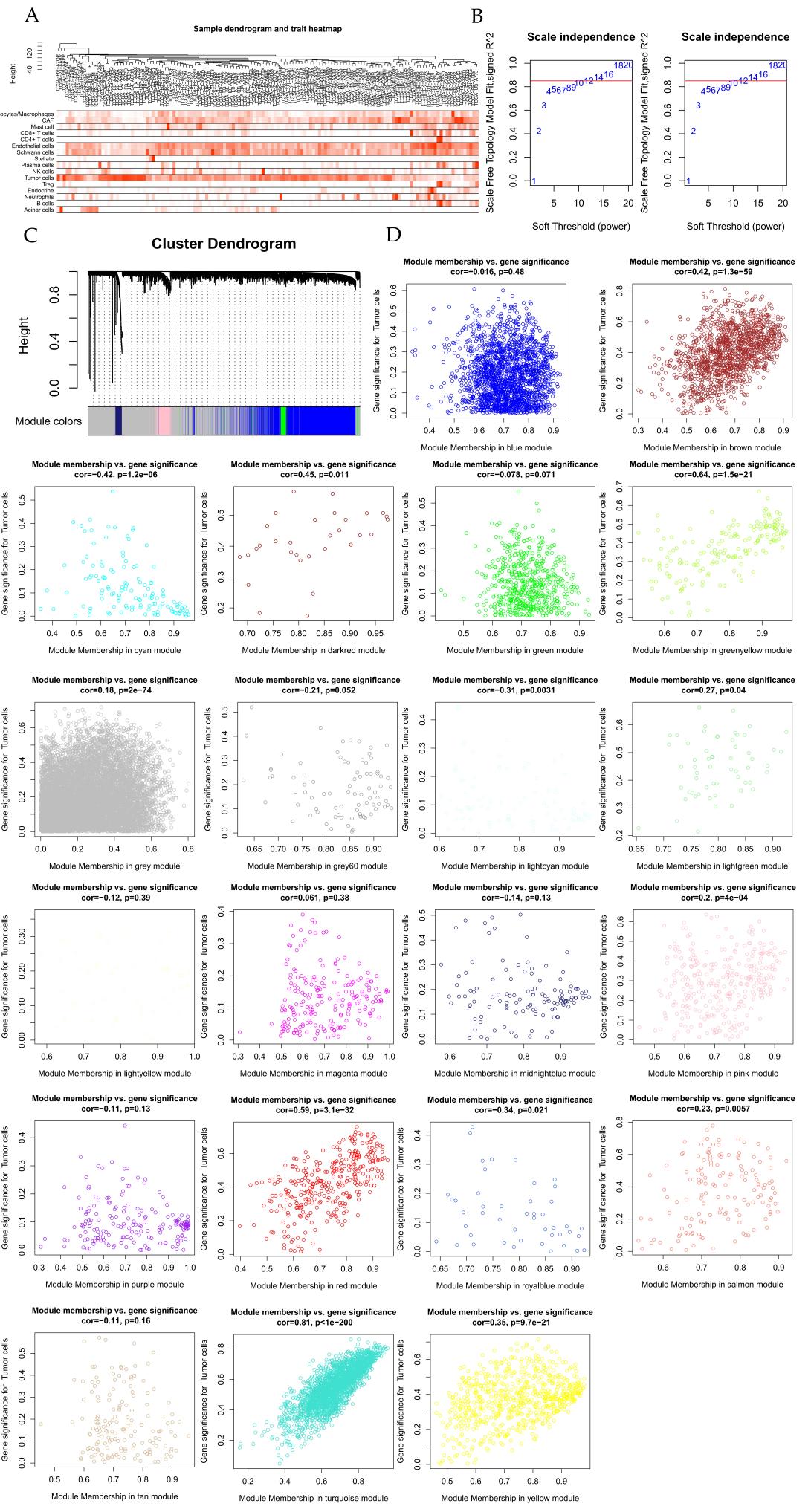


**Supplementary Figure 3.** WGCNA modules associated with tumor ductal cell abundance. **(A)** Sample clustering dendrogram based on whole-transcriptome expression, with an accompanying heatmap of clinical and cell-type traits (rows) used as external phenotypes, including estimated proportions of tumor ductal cells and other microenvironmental cell types. **(B)** Scale-free topology analysis across a series of soft-thresholding powers, showing the scale-free model fit and mean connectivity, respectively; the red dashed line marks the power selected for network construction. **(C)** Gene clustering dendrogram with corresponding module color bar, illustrating modules identified by dynamic tree cutting and subsequent merging. **(D**) Scatterplots of module membership (kME) versus gene significance for the Tumor cells trait for each color module. Each point represents a gene; titles report the Pearson correlation (cor) and P-value between kME and gene significance.


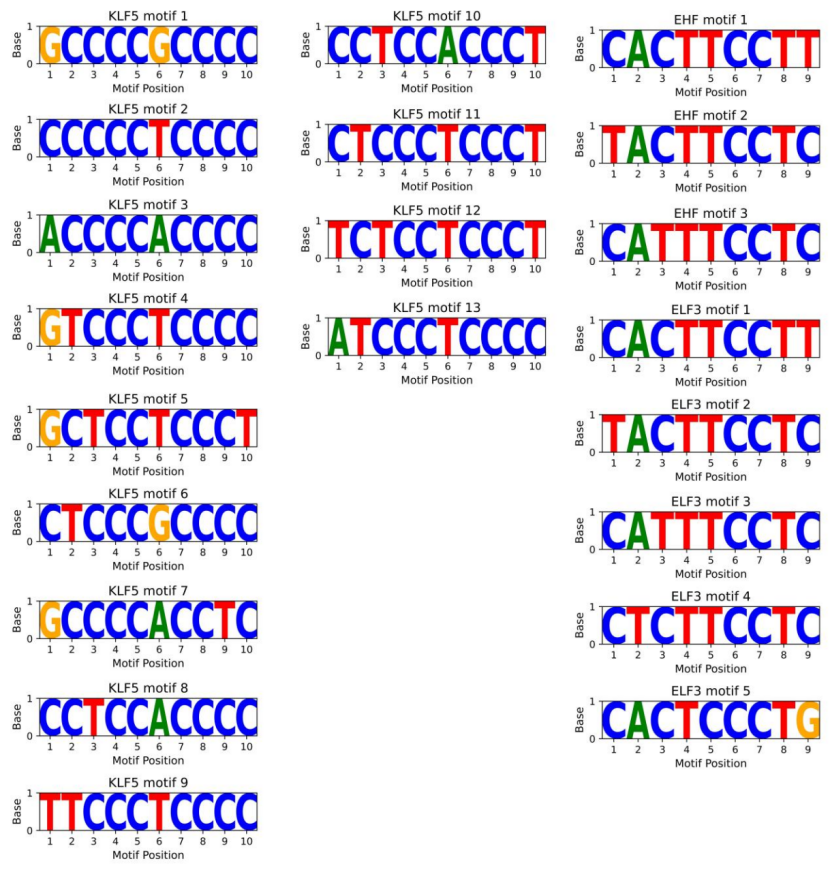


**Supplementary Figure 4.** Sequence logos of KLF5, EHF, and ELF3 binding motifs in the USP54 promoter. Representative DNA motif logos for KLF5, EHF, and ELF3 identified within the USP54 promoter region.


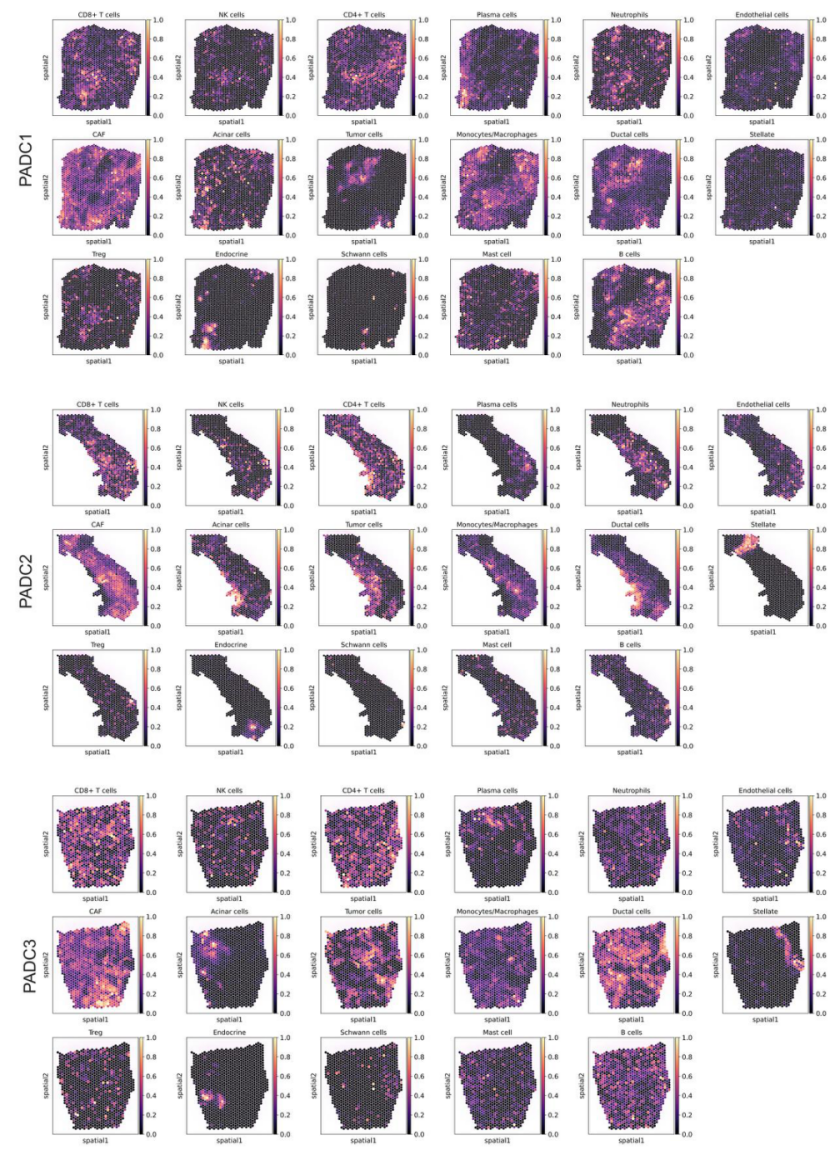


**Supplementary Figure 5.** Spatial distribution of inferred cell‐type abundances in three PDAC specimens. Heatmaps show the spatial localization of major immune and stromal populations in PDAC1, PDAC2, and PDAC3, as inferred from cell2location deconvolution. For each specimen, individual panels display normalized abundance scores (0-1, color scale) for CD8⁺ T cells, NK cells, CD4⁺ T cells, plasma cells, neutrophils, endothelial cells, CAFs, acinar cells, tumor cells, monocytes/macrophages, ductal cells, stellate cells, Treg cells, endocrine cells, Schwann cells, mast cells, and B cells across spatial coordinates (spatial1, spatial2). Brighter colors indicate higher predicted local abundance of the corresponding cell type.
